# A Machine Learning Approach to Predict Functional Performance From Measurable Protein Structural Characteristics: A Screening Tool for Protein Ingredient Quality

**DOI:** 10.1002/prot.70130

**Published:** 2026-03-11

**Authors:** Ronit Mandal, Sara Malvar, Ranveer Chandra, Baraem P. Ismail

**Affiliations:** ^1^ Department of Food Science and Nutrition University of Minnesota Saint Paul Minnesota USA; ^2^ Agricultural and Food Engineering Department, Indian Institute of Technology Kharagpur Kharagpur West Bengal India; ^3^ Microsoft Corporation, One Microsoft Way Redmond Washington USA

**Keywords:** functional properties, machine learning, plant proteins, regression

## Abstract

The food industry is witnessing the emergence of specialized protein‐based functional ingredients for the use as gelling, thickening, and/or emulsifying agents in various food applications. Different sources of protein including species and cultivars, as well as variable processing conditions affect the protein's structural characteristics, which in turn govern their functional properties. The complex relationship between the structure and function of the protein can be modeled using machine learning (ML) algorithms. In this study, different ML algorithms were used to predict solubility, emulsifying activity index, emulsifying capacity, and gel strength of different plant proteins using structural predictors (surface hydrophobicity, zeta potential, undenatured protein content, water holding capacity, soluble protein polymer content, *β*‐sheet content). Model performances were assessed by specific metrics ($R^{2}$, mean absolute error [MAE], and root mean squared error [RMSE]) and non‐violation of physical constraints. The solubility and emulsifying activity index were predicted using surface hydrophobicity, zeta potential, and undenatured protein content. Emulsifying capacity was predicted using surface hydrophobicity, solubility, undenatured protein content, while gel strength was predicted using solubility, undenatured protein content, water holding capacity, soluble protein polymer content, and *β*‐sheet content. The Gaussian based Support Vector Regression model accurately predicted solubility ($R^{2}$ = 0.8906), emulsifying activity index ($R^{2}$ = 0.7383), emulsifying capacity ($R^{2}$ = 0.7978), and gel strength ($R^{2}$ = 0.8822). Results highlighted the potential of ML algorithms for predicting of plant protein functionality using a few macromolecular structural characteristics. Such predictive models could serve as indispensable tools in the selection of protein ingredients for various food applications.

## 1 Introduction

The growing demand and dependency on plant proteins has led to steep market growth. The global plant protein market is expected to grow at a compound annual growth rate (CAGR) of 7.3% from 2022 to 2027 to reach a projected value of US $20.5 billion by 2029 [1]. This rampant growth is fueled by environmental and animal welfare concerns, as well as gravitation to healthy diets [2, 3].

Among the most common plant protein sources are soybeans and peas. In fact, soy protein is the most versatile and dominant among the plant proteins available in the market [1, 4, 5]. Emerging sources of protein include chickpea, camelina, pennycress, and hemp [6, 7, 8, 9, 10]. Plant protein ingredients such as isolates, concentrates, and hydrolysates are increasingly used as “clean label” functional ingredients in the formulation of different food products [11]. Functional properties such as solubility, gelling, water binding/holding, and emulsification allow plant proteins to be used in various products including beverages, baked goods, meat analogues, and dairy alternatives.

The aforementioned functional properties are dependent on various intrinsic (e.g., genotype, protein structure, profile, physicochemical characteristics) and extrinsic (e.g., pH, ionic strength, temperature) factors, processing (e.g., use of solvents, homogenization, extrusion), and protein interaction with other components [11]. Most plant proteins are comprised of water‐soluble albumins and salt‐soluble globulins, whose ratio and composition differ based on sources, Differences in the makeup of the different protein fractions determine the overall functional properties of plant proteins and thus their intended applications. Variation in the structural characteristics (e.g., molecular weight, amino acid composition, surface properties, configuration) contribute to differences in functional behavior [8, 12].

To understand the impact of varietal differences, processing, and formulation on the functional behavior of the protein, numerous characterization assays are performed [13, 14]. These assays are laborious, time consuming, expensive, require specialized equipment, and are often not standardized. While the impact of structural characteristics on functional behavior of proteins has been extensively studied [12, 15, 16, 17, 18, 19], direct and reliable prediction models are lacking. Developing data‐driven statistical models to correlate the complexity of the plant protein structure to its functional properties will aid in progressing the development of functional plant protein ingredients. Having such models in place allows for expanded experimental designs that encompass a large set of factors including many breeding lines or numerous levels of processing conditions.

Statistical models can be used to predict an output variable (such as protein solubility) based on the values of select input variables. Different statistical models using regression, response surface methodology, and partial least squares have been proposed [20, 21, 22]. For instance, Nakai [23] developed empirical equations based on linear regression to predict emulsifying activity index, emulsion stability, and oil binding capacity of milk proteins using surface hydrophobicity, solubility, and net surface charge. Similar models will allow the use of limited input variables such as surface hydrophobicity and solubility of a protein to numerically predict its emulsifying properties [24]. However, simple linear regression models often oversimplify the complex non‐linear nature of structure–function relationships. Traditional *in silico* approaches such as quantitative structure–activity relation (QSAR) modeling can predict protein function based on molecular characteristics like amino acid sequence and its native three‐dimensional structure [11]. Such QSAR modeling for prediction of specific protein's biological function in pharmaceutical research [25, 26] has not been employed in the food science field, since it is quite difficult to determine the protein amino acid sequence within a complex mixture such as plant protein isolates/concentrates. In addition, such protein systems undergo denaturation and conformational changes during processing, making prediction even more difficult [11] and QSAR modeling very complex. Therefore, in this study, the focus was to use a few macromolecular structural characteristics to predict different functional properties using various linear and non‐linear models, providing a more practical and data‐driven alternative for complex food protein systems.

The non‐linear relationships of plant protein structure–function could be explained by different machine learning (ML) algorithms. Several input variables (or predictors) could be potentially selected to adequately model non‐linear complex plant protein structure–function relation. This approach employing models can mathematically predict the functional properties for multiple plant protein species and should have validity and generalization over a large dataset. ML algorithms have been used to predict extensibility of extrudates during extrusion [27] and to predict how processing conditions affect the texture of yogurt [28]. Gel stiffness (Young's modulus) of pea ingredients was accurately predicted using artificial neural network (ANN) as a function of ingredients composition and processing history [29]. Arteaga and Nakai [30] used the ANN model to predict foaming and emulsifying properties of several food proteins. However, ANN are computationally intensive, have a black box nature (lack of interpretability), and overfit in case of limited data [31]. Also, these studies often explored small number of ML models, few functional properties, and relatively heterogeneous datasets. Therefore, the overall objective of this study is to develop prediction models for plant protein solubility, emulsifying properties, and gel strength using multiple ML models (ranging from linear and regularized models to tree‐based ensembles and kernel‐based methods) and a large set of analytical data produced under the same experimental conditions. Such approach highlights the novelty of this work. ML models' acceptability was assessed based on their statistical performance metrics as well as non‐violation or conformation to physical constraints (unrealistic minima or maxima or ≥ 0 values).

## 2 Materials and Methods

### 2.1 Dataset Preparation and Preprocessing

For any good quality modeling, good quality data is the most important requirement. The dataset used in this study was compiled by combining the analytical data from the research works done at Plant Protein Innovation Center, University of Minnesota on different plant protein species, viz. soy ( *Glycine max* ), pea ( *Pisum sativum* L.), chickpea ( *Cicer arietinum* L.), rice ( *Oryza sativa* L.), hemp ( *Cannabis sativa* L.), camelina ( *Camelina sativa* L.), and pennycress ( *Thlaspi arvense* L.). The aim was to include different plant proteins to build a robust and accurate predictive model that can explain variations in the dataset arising out of source irrespective of product type, extraction conditions, or pretreatment; and predict functionality for unseen, new data. Soy protein samples used in the study were produced from dehulled beans that were flaked, defatted, and subjected to alkaline protein extraction (pH 7.5–8.5) to obtain isolates [15]. Soy protein concentrates were produced by wet milling and obtained from a commercial source. Pea and chickpea protein isolates were produced following a similar wet extraction process to that used to obtain soy protein isolates [15, 19], while their concentrates counterparts were produced by air classification and obtained from commercial sources. Rice protein isolates were obtained from a commercial source. Hemp protein isolates were produced following alkaline (pH 11) or salt extraction (0.75 M NaCl at 50°C) [7]. Camelina and pennycress protein isolates were produced following alkaline (pH 11 in 0.1% (w/v) sodium sulfite solution) or salt extraction [9]. Protein content was determined following the Dumas method (AOAC 990.03) using a LECO FP828 nitrogen analyzer (LECO, St. Joseph, MI, USA) and a conversion factor of 6.25. Isolates contained > 80% by weight protein, concentrates had 50%–60% by weight protein and flour < 30% by weight protein.

All the protein samples were evaluated based on different structural characteristics including surface hydrophobicity using fluorometric ANS probe method, zeta potential using dynamic light scattering instrument, denaturation enthalpy using differential scanning calorimetry, molecular weight distribution of soluble protein polymers, and functional proteins (7S, 11S globulins) using size exclusion high performance liquid chromatography, and protein secondary structure using attenuated total reflectance Fourier‐transform infrared spectroscopy [32]. For model development, five structural features (surface hydrophobicity [SH], zeta potential [ZP], % undenatured protein [U] Equation 1, soluble protein polymers [SP], and *β*‐sheet % [βs]) were selected. The functional properties measured were protein solubility (Sol) [%] at pH 7.0, emulsifying activity index (EAI) [m^2^/g], emulsifying capacity (EC) [g/g], water holding capacity (WHC) [%], and gel strength (Gel) [N] [32, 33]. All the analytical procedures used for the analysis were performed under the same experimental conditions.

(1) $$U\;\left[\%\right]=\frac{∆H}{∆H_{n}}\times 100$$

where ∆H = Denaturation enthalpy of test protein sample [J/g]; $∆H_{n}$ = Denaturation enthalpy of native protein sample [J/g].

The plant protein dataset, which included samples grouped by sources along with their functional properties and structural characteristics, was prepared as a spreadsheet (Microsoft Excel) for further data analysis. The dataset (150 data points) was checked for missing values and non‐numeric characters. Categorical variables were transformed into coded variables for analysis. No data normalization or standardization was carried out for data preprocessing.

### 2.2 Data Visualization and Principal Component Analysis

Prior to the implementation of ML model on the input data, it was crucial to determine the relationships among the set of variables. Therefore, a preliminary exploratory data visualization was conducted to investigate the associations between the functional parameters (dependent variables) and different structural characteristics (independent variables). The analysis aimed to gain knowledge about the inherent patterns, possible dependencies, and linear or non‐linear relationships among the variables within the dataset, which provided the foundation for the subsequent ML modeling. To facilitate data visualization, scatterplots of the data were generated using R version 4.2.22022.07.1 + 554 running on a Windows 10 system. RStudio is an integrated development environment for R that provides comprehensive tools for data analysis and modeling. Scatterplots were generated using the “ggplot2” package of RStudio.

Multivariate data analysis was performed using principal component analysis (PCA) on the dataset to explore the structure and patterns in the data. For standard PCA algorithm implementation, “prcomp” package of RStudio was used, which is based on singular value decomposition technique that examines the covariances/correlations between individuals. Prior to PCA, the data was centered and scaled to make it comparative, to capture relative variations, and to overcome biases. PCA enabled the identification of principal components that can explain > 60% of the variances in the dataset.

### 2.3 Machine Learning Algorithm Implementation

The present study aimed to explore and numerically predict the values of dependent variable (Y) (functional properties) as a function of input variables (X) or predictors (structural characteristics). Such an approach was achieved by employing a suite of machine learning models capable of capturing the quantitative relationships between structural characteristics and functional properties across diverse plant protein species, ensuring broad applicability and generalization. Predictor, X was a set of variables: SH, ZP [mV], U [%] (Equation 1), SP [%] and βs [%] (Equation 2). The dependent variable, Y were Sol [%], EAI [m^2^/g], EC [g/g], and Gel [*N*] (Equation 3).

(2) $$X=\left\{\mathrm{SH},\mathrm{ZP},U,\mathrm{SP},\mathrm{\beta s}\right\}$$

(3) $$Y=\left\{\mathrm{Sol},\mathrm{EAI},\mathrm{EC},\mathrm{Gel}\right\}$$

Individual Y variables were expressed as a function of selected predictors X, based on domain knowledge on which variables affect them most. Sol and EC were predicted using SH, ZP, and U (Equations 4 and 5, respectively). EAI was predicted based on SH, U. Considering the observed strong correlation between EAI and Sol (Pearson's correlation coefficient, *r* = 0.744; *p* < 0.0001), Sol was incorporated into the predictive model (Equation 6). Gel was predicted using variables Sol, WHC (EAI~WHC: r = 0.475; *p* = 0.0000), βs, SP, U (Equation 7). βs content was included in the model as it has been shown to positively contribute to the gelation properties of proteins [17, 32, 34, 35].

(4) $$\mathrm{Sol}=f\left(\mathrm{SH},\;\mathrm{ZP},\;U\right)$$

(5) $$\mathrm{EC}=f\left(\mathrm{SH},\;\mathrm{ZP},\;U\right)$$

(6) $$\mathrm{EAI}=f\left(\mathrm{SH},\;\mathrm{Sol},\;U\right)$$

(7) $$\mathrm{Sol}=f\left(\mathrm{Sol},\;\mathrm{WHC},\;U,\;\mathrm{SP},\;\mathrm{\beta s}\right)$$

Thirteen different ML models were then trained on the input data to build predictions using RStudio version 4.2.2. Model types ranged across both linear and non‐linear algorithms, allowing for a robust comparison of predictive performance and interpretability. Linear and Poisson regression models were studied to capture simple linear relationships between variables. Polynomial, spline, and log‐linear regression models were used to explore moderate nonlinearities. Ensemble models like Random Forest, gradient boosting machine (GBM), and Decision Tree were used for their strong performance in capturing complex feature interactions. Support vector machine regression (SVR) and Gaussian SVR were studied due to their ability to use kernel functions to transform data into high‐dimensional space. Neural Networks were used for their ability to identify non‐linear trends in the data. K‐Nearest Neighbors (k‐NN) and Lasso Regression were employed as non‐parametric, instance‐based learning algorithm and feature regularization, respectively. Firstly, a multiple linear regression model was used to analyze the linear relationship between variables X~Y, using R package stats (version 4.2.2), as given by the general form (Equation 8).

(8) $$Y=\beta_{0}+\beta_{1}X_{1}+\beta_{2}X_{2}+\ldots +\beta_{n}X_{n}+\varepsilon$$

where $X_{1}$, $X_{2}$, …, $X_{n}$= independent variables; $\beta_{0}$ = intercept; $\beta_{1}$, $\beta_{2}$, …, $\beta_{n}$ = Coefficients associated with the independent variables; ε = Residual error term.

Polynomial regression model was used, in which the individual predictors were raised to *n*th degree (n = 1–5), to capture the inherent non‐linear relation between X~Y that can better fit the complex dataset. The general form of the model is shown in Equation (9).

(9) $$\begin{array}{c} Y=\beta_{0}+\beta_{11}X_{1}+\beta_{12}X_{1}^{2}+\ldots +\beta_{1n}X_{1}^{n}+\beta_{21}X_{2}+\beta_{22}X_{2}^{2}\\ \;+\ldots +\beta_{2n}X_{2}^{n}+\ldots +\beta_{\mathrm{mn}}X_{m}^{n}+\varepsilon \end{array}$$

where $X_{1}$, $X_{2}$, …, $X_{n}$= independent variables; $\beta_{11}$, $\beta_{12}$, …, $\beta_{\mathrm{mn}}$ = Coefficients associated with the independent variables. The model parameter n can vary for individual variables, to allow capturing non‐linearity in the dataset. The R package stats was used for the polynomial model.

Log‐linear model was used by transforming the expected values of Y ($E\left(Y\right)$) on a logarithmic scale, to study the complex relationships and interactions between variables that were missed by the linear model. The Log‐linear model (Equation 10) was implemented using glm package.

(10) $$\log\left[E\left(Y\right)\right]=\beta_{0}+\beta_{1}X_{1}+\beta_{2}X_{2}+\ldots +\beta_{n}X_{n}+\varepsilon$$

The Poisson regression model, a specific instance of the log‐linear framework, was applied, under the assumption that the response variable *Y* adheres to a Poisson distribution (where mean equals variance), making it suitable for count data and rare event modeling. This model is particularly effective in scenarios where the response variable represents discrete occurrences across fixed intervals or areas.

Lasso (Least Absolute Shrinkage and Selection Operator) regression model, based on L1 regularization technique, was employed, leveraging an L1 penalty to enforce sparsity in the coefficient estimates, thereby reducing model complexity and enhancing interpretability by shrinking some coefficients to zero. This model was implemented using the “glmnet” R package version 4.1.7.

Spline model, which employs piecewise polynomial functions (splines) $f\left(x\right)$ was utilized to model non‐linear relationships by fitting piecewise polynomials, allowing for flexibility in capturing local variations and abrupt changes in the data. The splines were fitted using local least squares, enabling detailed representation of complex patterns. Each of the smaller splines were fitted using the local least square method, which allowed greater flexibility and provides accurate representation of X~Y. The general form of the spline model is given in Equation (11).

(11) $$Y=\beta_{0}+\beta_{11}X_{1}+\beta_{12}X_{1}^{2}+\ldots +\beta_{\mathrm{mn}}X_{m}^{n}+f\left(x\right)$$

Decision tree algorithm, characterized by a hierarchical tree structure of nodes and branches, was evaluated for its ability to segment the dataset based on decision rules derived from the input features. This model was implemented using the “rpart” package version 4.1.19. Each node allows a decision to be made that further divides the data into branches, which yield leaf nodes that result in a prediction. The decision tree algorithm was applied using rpart version 4.1.19. Decision trees often suffer from overfitting, which was mitigated by \using ensemble methods like random forest or adjusting the hyperparameters, such as tree depth.

Random forest model includes an ensemble of multiple decision trees for accurate and robust predictions. The algorithm creates an ensemble of trees, where each tree evaluates a subset of bootstrapped samples from the dataset and the predictor variables *X*. Random Forest algorithm was applied using the randomForest R package version 4.7.1.1.

Gradient boosting machine (GBM), which is an ensemble of decision trees, was applied. GBM combines the predictions of multiple weak learners (decision trees) resulting in a gradient‐boosted tree. This gradient‐boosted tree sequentially trains the model based on previous model in the ensemble. GBM algorithm was applied using the gbm R package version 2.1.8.1.

Support vector machine (SVM) based regression model was used for prediction modeling of relation X~Y. This model was applied to map input features into high‐dimensional space using kernel functions, aiming to find a hyperplane that best separates the data with a margin defined by the epsilon (ϵ) parameter. In the model, the datapoints that were bound in the ϵ‐tube are called support vectors. The general form of the SVM model is given in Equation (12).

(12) $$Y=w^{T}\phi \left(X\right)+b$$

where $w^{T}$= Transpose of weight vector (vector containing the weights assigned to each transformed feature); $\phi \left(X\right)$ = Feature transformation of input variables based on the applied kernel function; b = Bias term. Feature transformation of the SVM model was done using a linear kernel function employing the *vanilladot* function of the R package kernlab version 0.9.32 (Equation 13).

(13) $$K\left(x,x^{\prime }\right)=x\cdot x^{\prime }$$

*Gaussian* model, another type of SVM (called *Gaussian* based Support Vector Regression) was used for modeling X~Y. Feature transformation mapping of the input variables to high‐dimensional space in the *Gaussian* model was carried out using the kernel Radial Bias Function *rbfdot* (Equation 14) using the R package kernlab version 0.9.32.

(14) $$K\left(x,x^{\prime }\right)=\exp\left(-\gamma \times {\left\|x-x\prime \right\|}^{2}\right)$$

where $x,x^{\prime }$ = input feature vectors; ${\left\|x-x\prime \right\|}^{2}$ = Squared *Euclidean distance* between x and $x^{\prime }$; γ = width parameter for *Gaussian* curve ($\frac{1}{\sigma^{2}}$).

The k‐Nearest Neighbors (k‐NN) model algorithm was also evaluated. k‐NN model is a non‐parametric method of predicting the values of Y based on the values of its k neighbors in the feature space. The k‐NN model in simple form is given in Equation (15), which denotes the mean value of the predicted variable Y form its *k* neighbors. The k‐NN model was employed using R package class version 7.3.22 based on *Euclidean distance* metric to calculate the distance of new datapoint and training datapoints.

(15) $${\hat{y}}_{i}=\frac{1}{k}\sum_{i=1}^{k}y_{i}$$

where ${\hat{y}}_{i}$ = Predicted value for the new data point; $y_{i}$= Observed values of the target variable for the i‐th nearest neighbor; k = number of nearest neighbors considered.

Lastly, a neural network (NN) model was fitted on the dataset using a backpropagation technique. The NN model contained several hidden layers between the input and output layers, which have several interconnected nodes (neurons). Sigmoid activation function ($f\left(x\right)=\frac{1}{1+\exp\left(-x\right)}$) was used to train the model to learn complex relationship in the dataset. The NN model was run using R package nnet version 7.3.19 or neuralnet version 1.44.2.

#### 2.3.1 Model Training and Cross‐Validation Technique

The input dataset in Excel spreadsheet format was analyzed using RStudio software (version 4.2.2) with appropriate ML algorithm packages described earlier. All analyses were performed on a Windows 10 Dell system equipped with 16 GB RAM, using CPU processing only (no GPU acceleration). The computational demand was minimal, and all analyses can be readily reproduced on a standard personal computer. The whole dataset was randomly split into two sets (*Train* set and *Test* set) in the ratio 70% (*Train*) and 30% (*Test*) using the R program. Model training was typically done on the labeled *Train* data where different models learn the patterns and relationships in the data. During the model training, the model parameters were adjusted to minimize the differences between the actual and predicted variables. The performance of the model was then assessed on the unseen *Test* data, which was separate from the *Train* data. Evaluation of model performance based on the independent dataset estimated how well the model predicted and generalized for an unseen, new data.

Cross‐validation of the model was also performed to assess how well the model generalized for unseen data. Cross‐validation assisted in evaluating robustly how well a particular model generalized the prediction in the case of unseen data and took care of issues like model overfitting or underfitting. For this method, a k‐fold cross‐validation technique was adopted for the training set whereby the set was iteratively split into 5 equal‐sized subsets or folds, and models were iteratively fitted on the folds every time. The model was trained and tested on different combinations of these folds, allowing for a more comprehensive evaluation. The model metric and performance were then evaluated for all the five folds and then averaged. The best model was chosen based on low cross‐validation error.

The overall workflow of the ML based predictive modeling of plant protein functionality was: (1) Creating the plant protein dataset for different proteins and their structural‐functional data, (2) Train the mentioned ML models, and (3) Evaluate the performance metric of models and their conformation to real physical constraints. The complete code of the ML modeling on the dataset can be obtained from Open Code on the GitHub website https://github.com/ronit09mandal/ML‐Prediction‐of‐Plant‐Proteins‐Functionality/tree/main.

#### 2.3.2 Hyperparameter Tuning

Each trained ML model included hyperparameters, which are not directly learned from the data but instead govern the models' predictive capabilities. These hyperparameters influence the behavior and complexity of the models and require optimization to prevent overfitting. The hyperparameters controlled the behavior and complexity of the models and needed to be optimized or tuned to control overfitting to the data. Model hyperparameters were optimized using grid search and k‐fold cross‐validation (*k* = 5) following standard tuning practices as described by Claesen and De Moor [36] and Ippolito [37]. For polynomial model, the degree n (1–5) was tuned for lowest root‐mean squared error (RMSE) and each of the predictor X was adjusted to be raised to different degree n, if applicable. Higher degree n controls the fitting to complex data but may overfit the data. The polynomial model was subjected to L1 regularization to yield log‐linear and Poisson models. The hyperparameters, α and λ, controlled the regularization strength and model complexity, and were thus tuned. Lasso regression was a specific case of Poisson model where α = 1. The spline model, an enhancement over the polynomial model, decomposed the function into smaller polynomial segments or regions called knots, enabling it to fit complex data more effectively. The hyperparameter (number of knots for each predictor X) was tuned for best RMSE value. Decision tree model was comprised of nodes (root, internal, leaf) and branches. The decision tree model consisted of nodes (root, internal, leaf) and branches. The model's hyperparameters included maximum depth (number of levels or splits), minimum sample split (the minimum number of samples required to split internal nodes), and minimum sample leaf (the minimum number of samples in a leaf node). In case of random forest algorithm, the number of features selected (mtry) for each node was tuned. The GBM model was tuned controlling the hyperparameters—number of trees (n.trees), learning rate or shrinkage and interaction depth. For the SVM model with linear or radial bias kernel function, the cost parameter C was tuned. For *Gaussian* based Support Vector Regression, hyperparameter γ that controlled the width of *Gaussian* curvature was also tuned. The hyperparameter k in case of k‐NN model, which controlled the number of nearest neighbors, was optimized. In the case of neural network model, hyperparameters, like number of hidden layers and number of neurons in each layer, were optimized.

#### 2.3.3 Model Performance and Selection

Model performance for the selection of best performing model was conducted based on the known statistical metrics as well as non‐conformity to physical constraints (unreal maxima/minima and ≥ 0 values). For the statistical metrics, Pearson's correlation coefficient, r was calculated between observed analytical values of Y ($y_{i}$) and the predicted values of Y (${\hat{y}}_{i}$). Additionally, the coefficient of determination, $R^{2}$ was computed to quantify the proportion of variance in the observed data explained by the model. Error metrics between $y_{i}$ and ${\hat{y}}_{i}$ including mean absolute error (MAE) and root mean square error (RMSE), were calculated as follows (Equations 16 and 17).

(16) $$\mathrm{MAE}=\frac{1}{n}\sum_{i=1}^{n}\left|y_{i}-{\hat{y}}_{i}\right|$$

(17) $$\text{RMSE}=\sqrt{\frac{1}{n}\sum_{i=1}^{n}\left(y_{i}-{\hat{y}}_{i}\right)}$$

In addition, the conformation of the models to physical behavior (no unrealistic maxima or minima or ≤ 0 values of predicted Sol, EAI, EC and Gel) were considered based on domain knowledge. For Sol, only models with values ranging from 0% to 100% protein solubility were selected, since this is the physically possible range of solubility. For EAI, EC and Gel, values ≥ 0 units were selected, with no maxima. A similar model feasibility approach was adopted by researchers previously [29, 38].

## 3 Results and Discussion

### 3.1 Data Visualization and Principal Component Analysis

**FIGURE 1:**
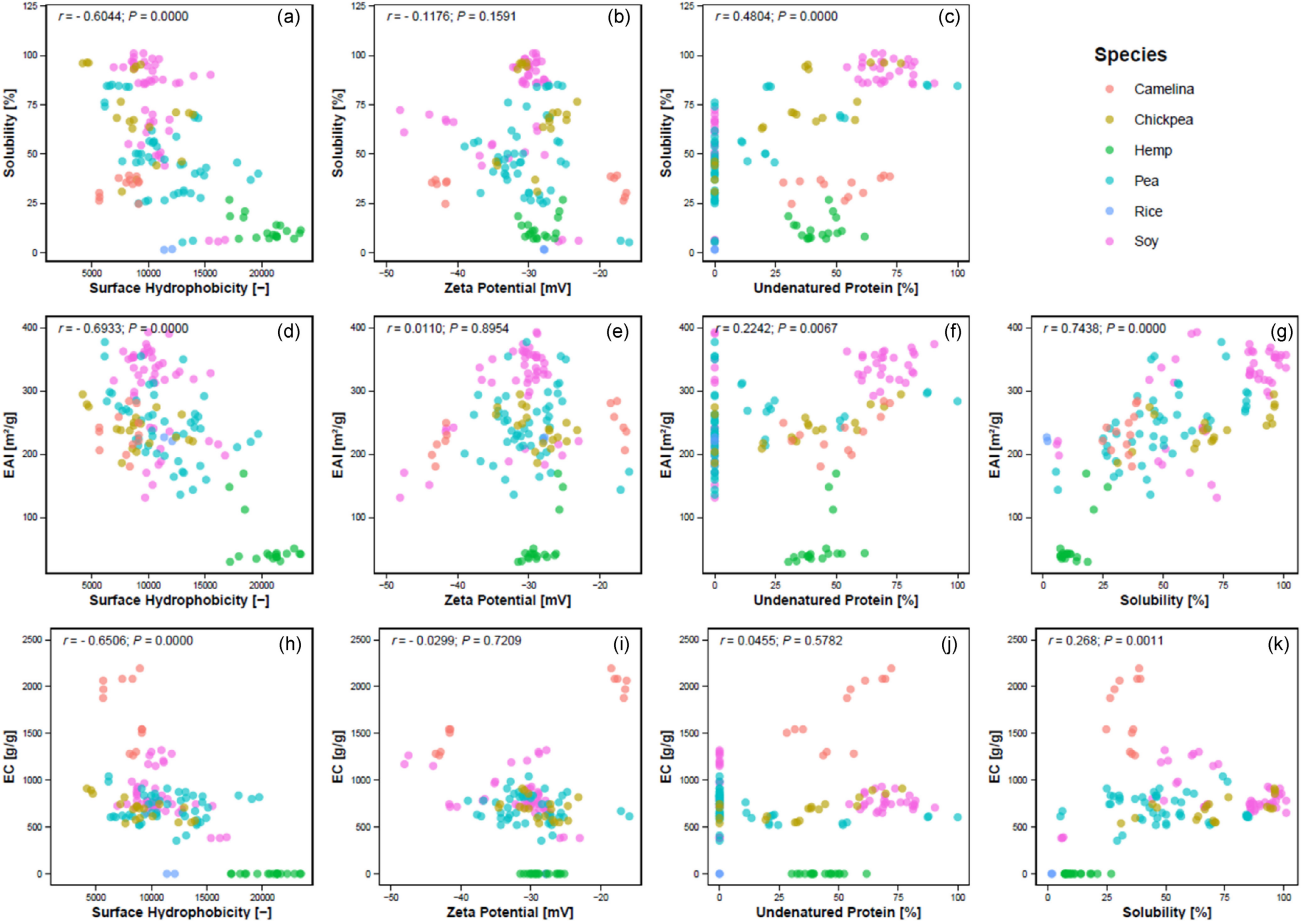
Scatterplots for plant protein solubility (pH 7.0) (𝑆𝑜𝑙), emulsifying activity index (𝐸𝐴𝐼) and emulsifying capacity (𝐸𝐶) versus surface hydrophobicity (a, d, h), zeta potential (b, e, i), undenatured protein (c, f, j), and emulsifying activity index and emulsifying capacity versus solubility (g, k). Individual species of proteins are color coded as per legend.

**FIGURE 2:**
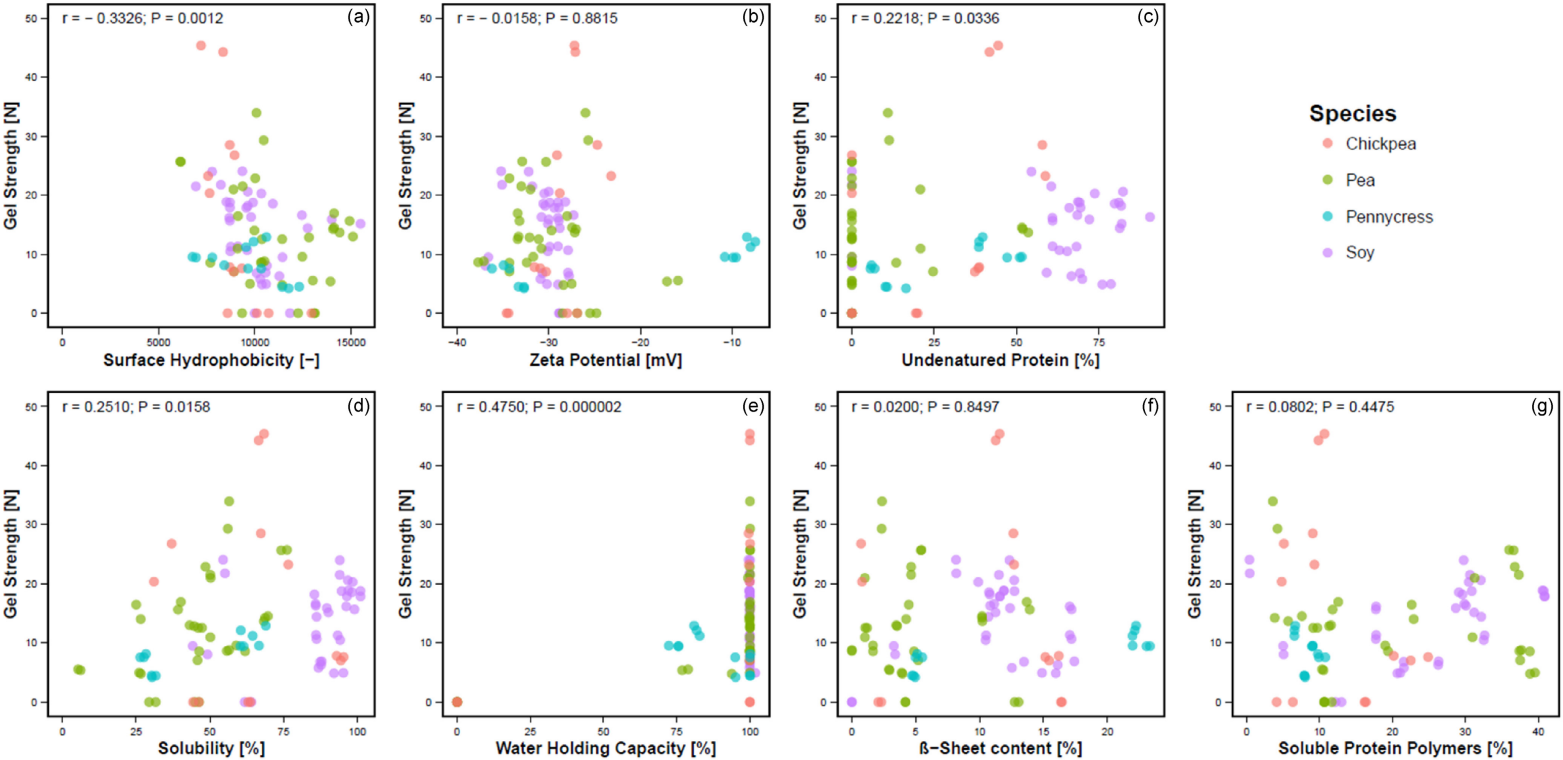
Scatterplots for plant protein gel strength (𝐺𝑒𝑙) versus surface hydrophobicity (a), zeta potential (b), undenatured protein (c), solubility (pH 7.0) (d), water holding capacity (e), *β*‐sheet content (f), and soluble protein polymers content (g). Individual species of proteins are color coded as per legend.

Data visualization using scatterplots is a powerful tool for understanding and analyzing complex relationships, such as those of plant protein functionality and structure. For instance, a scatterplot can show how protein functionality is distributed among different protein sources, which formed the basis of modeling plant protein relationships X~Y, as described in Section 2.3. Figures 1 and 2 show the scatterplots for the plant protein analytical data with the annotations of the calculated Pearson's correlation coefficient (r) and the probability value (*p*‐value). The protein functional properties, Sol, EAI, ES, Gel variables, were expressed as a distribution of the set of independent variables, SH, ZP, U, SP, and βs. A moderately strong negative correlation was observed between Sol and SH (Figure 1a), indicating that SH of plant proteins strongly govern their solubility. Proteins with higher SH have a natural tendency to aggregate due to hydrophobic interactions, leading to lower solubility [39]. Rice and hemp protein samples, which had the highest SH, demonstrated the lowest solubility (<25%), while soy proteins showed the highest solubility (up to 100%). Additionally, a moderately strong positive correlation (Figure 1c) was observed between Sol and U, indicating that a high level of native undenatured proteins contributed to high solubility. The Sol and ZP relationship was less evident, as was that of EAI versus ZP and U (Figure 1e,f). Emulsifying properties are particularly described by stabilization and activity of proteins at the interface. EAI was better correlated to SH and Sol, with strong negative and positive correlations, respectively (Figure 1d,g). This correlation indicated that better surface activity of proteins as represented by a higher EAI is attributed to lower SH and higher Sol. While EC strongly correlated with SH as EAI did, it weakly correlated with Sol. High protein solubility often contributes to reduced interaction with the oil phase, limiting the capacity of the protein to emulsify the oil [40, 41].

The gel strength, Gel, ranged from 0 to 45 N, with the highest values for chickpea and lowest for pea. Gel showed weak, negative correlations with SH, U and Sol, while it had a moderately strong positive correlation with WHC (Figure 2a–e). Other variables, including U, βs, and SP weakly correlated with Gel. However, the interaction of those variables could be relevant in governing Gel of different proteins. Computational modeling, derived out of ML algorithms, was thus employed to account for such complex interactions within the protein sample dataset.

**FIGURE 3:**
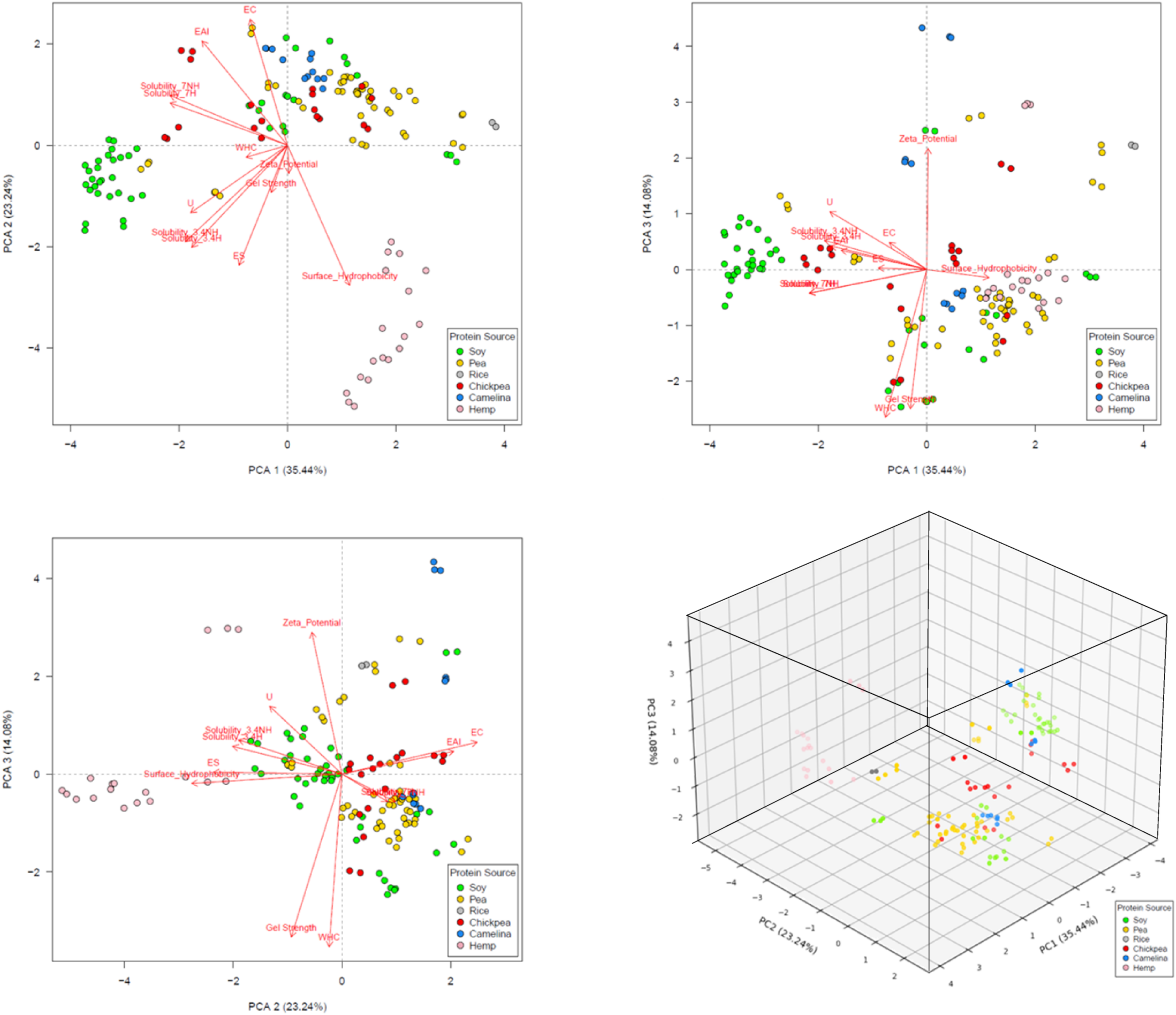
Principal component analysis of the first three components that represents over 72% of the variance at 95% confidence. Individual species of proteins are color coded as per legend.

PCA was used to explore the relationships among the analytical data of structural characteristics and functional properties across a diverse set of different plant proteins, which was standardized to ensure that all properties contributed equally to the analysis (Figure 3). While the first three principal components (PCs) are shown in Figure 3, PC1 and PC2 accounted for the highest proportion (~60%) of the total variance, explaining 35.44% and 23.24% of the variability, respectively.

The variable axes for Sol and SH fell on opposite quadrants (Figure 3), confirming that they were negatively correlated, as was evident in Figure 1a. Gel and WHC loading axes lied closer to each other showing a positive correlation and a close association. The scores of the soy protein samples lied closer to the loading axis of Sol, confirming high solubility. On the other hand, the scores of the hemp protein samples fell closer to SH loading axis, confirming their high SH and low Sol, and therefore poor functionality. Soy protein scores also lied closer to the axes of EAI, EC and Gel, indicating better functional properties compared to other proteins. The scores of chickpea and camelina proteins indicated good emulsifying properties since they lied near the loading axes of EAI and EC. The scores of pea proteins lied in the opposite quadrant to that of Gel, confirming their poor gelling property. Rice proteins' poor functional properties were evident based on the scores that lied far from the loading axes for all the variables. Rice proteins contain a huge number of intra‐ and intermolecular disulfide bonds, have a high extent of hydrophobic interactions, and are characterized by a rigid globular structure, limiting their molecular flexibility and imparting poor functionality [42].

Observations confirmed that PCA allowed separation of protein sources based on their structural characteristics and functional properties. Proteins that were in the same cluster exhibited similar structural characteristics and functional properties. A substantial amount of information was effectively captured and represented in a lower‐dimensional space using PCA, which formed the basis for further ML modeling.

### 3.2 Predictive Modeling of Plant Protein Functionality

Different ML algorithms including linear, log‐linear, lasso, polynomial, gradient boosting machine, Poisson, support vector regression, *Gaussian* based Support Vector Regression, spline, decision tree, random forest, neural network, k‐nearest neighbors, were all evaluated based on their data fitting ability and prediction of protein functionality Y. The models were developed considering the independent variables (X) SH, ZP, U, βs, Sol, and SP. All the hyperparameters were tuned for the trained models (Table S1).

#### 3.2.1 Prediction of Protein Solubility

**FIGURE 4:**
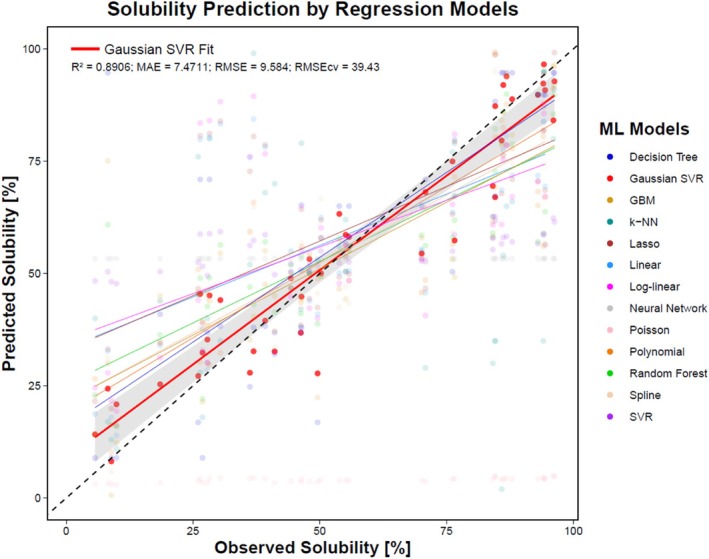
Actual versus predicted protein solubility (pH 7.0) (Sol) plot based on fitting different machine learning (ML) models. Individual models are color coded as per legend. MAE, mean absolute error; RMSE, root mean squared error.

The scatterplot for actual ($Y_{i}$) versus predicted (${\hat{Y}}_{i}$) Sol using the various ML prediction models is shown in Figure 4. The model parameters, significance values of coefficients, RMSE, MAE and $R^{2}$ for the models with tuned hyperparameters are given in Table 1. Substantial differences were observed in the predictive performance of the evaluated 13 ML algorithms for the different plant protein sources. The actual variations in protein Sol values are shown as box plot distributions in Figure S1a. With the lowest MAE and RMSE, and highest $R^{2}$, *Gaussian* based Support Vector Regression model showed the best fit for the overall dataset. The prediction capability of the Poisson model was the poorest (Figure 4), with high prediction errors (RMSE and MAE) (Table 1). The Poisson model under‐estimated all the data points with a mean prediction value of 4.043%. The neural network model also had poor prediction, with a mean Sol of 53.33% for all observations. Similarly, the SVM model performed poorly with a mean prediction for Sol of 55.03%. Hence, these models did not properly predict the Sol of the proteins.

**TABLE 1:** Machine learning models comparison for protein solubility: Model parameters and performance metrics.

Model	Model parameters	Performance metrics
Linear Regression	Coefficients: Intercept = 62.69 (*p* = 9.050e‐06·***) Surface Hydrophobicity = −0.0039 (*p* = 3.860e‐14·***) Zeta Potential = −0.7632 (*p* = 0.0334·*) Undenatured protein = 0.4987 (*p* = 2.390e‐12·***) Residual standard error: 18.84 on 100 degrees of freedom Multiple *R* ^2^ = 0.6182, Adjusted *R* ^2^ = 0.6068	MAE = 17.19 *r* = 0.6809 *R* ^2^ = 0.4636 RMSE = 22.36 *p* < 2.200e‐16
Polynomial regression	Coefficients: Intercept = 53.33 (*p* < 2.000e‐16·***) Surface Hydrophobicity = −171.4 (*p* = 2.900e‐15·***) (Surface Hydrophobicity)^2^ = −45.94 (*p* = 0.0187·*) (Surface Hydrophobicity)^3^ = 0.1523 (*p* = 0.9929) Zeta Potential = −40.81 (*p* = 0.0233·*) (Zeta Potential)^2^ = −71.48 (*p* = 7.080e‐05·***) (Zeta Potential)^3^ = −35.92 (*p* = 0.0397·*) Undenatured protein = 155.4 (*p* = 5.000e‐13·***) (Undenatured protein)^2^ = 25.13 (*p* = 0.1962) (Undenatured protein)^3^ = −13.04 (*p* = 0.4459) Residual standard error: 16.80 on 94 degrees of freedom Multiple *R* ^2^ = 0.7146, Adjusted *R* ^2^ = 0.6873	MAE = 11.22 *r* = 0.8686 *R* ^2^ = 0.7545 RMSE = 14.25 *p* < 2.200e‐16
Random forest	Tree structure: 500 trees % Variable explained = 74.85 Importance (Increase in Node Purity): Surface Hydrophobicity = 29158.7 Zeta Potential = 18327.6 Undenatured protein = 38959.4 Mean of squared residuals: 224.9	MAE = 12.80 *r* = 0.8163 *R* ^2^ = 0.6663 RMSE = 17.04
Support vector machine regression (SVR)	Support vectors: SV type: Epsilon‐svr (regression) Number of Support Vectors = 100 Epsilon = 0.1 cost C = 0.1 Gamma = 0.01 Kernel parameters Linear (vanilla) kernel function Objective function value = −7.419 Training error = 0.8067	MAE = 23.03 *r* = 0.6613 *R* ^2^ = 0.4373 RMSE = 26.11
Gaussian SVR	SV type: Epsilon‐svr (regression) Number of support vectors = 75 Epsilon = 0.1 cost C = 10 Gamma = 1 Gaussian Radial Basis kernel function Hyperparameter: sigma = 1 Objective function value = −78.70 Training error = 0.0513	MAE = 7.4711 *r* = 0.9437 *R* ^2^ = 0.8906 RMSE = 9.584 RMSE_CV_ = 39.43
Neural network	Network architecture: Hidden layers: 7, 5 Activation function: Logistic: $f\left(x\right)=\frac{1}{1+e^{-x}}$	MAE = 24.99 *r* = _ *R* ^2^ = _ RMSE = 28.70
Spline regression	Coefficients: Intercept = 63.10 (*p* < 0.000585·***) Surface Hydrophobicity = −9.139 (*p* = 0.5041) (Surface Hydrophobicity)^2^ = −23.86 (*p* = 0.0555.) (Surface Hydrophobicity)^3^ = −72.64 (*p* = 0.0150·*) (Surface hydrophobicity)^4^ = −76.42 (*p* = 1.410e‐10·***) Zeta potential = 11.63 (*p* = 0.2943) (Zeta potential)^2^ = −7.666 (*p* = 0.5215) (Zeta potential)^3^ = 6.195 (*p* = 0.6334) (Zeta potential)^4^ = −45.36 (*p* = 0.0980.) (Zeta potential)^5^ = −42.40 (*p* = 0.0006·***) Undenatured protein = 18.02 (*p* = 0.1570) (Undenatured protein)^2^ = 45.28 (*p* = 1.330e‐4·***) (Undenatured protein)^3^ = 42.66 (*p* = 8.760e‐07·***) Residual standard error: 16.56 on 91 degrees of freedom Multiple *R* ^2^ = 0.7317, Adjusted *R* ^2^ = 0.6963	MAE = 13.10 *r* = 0.8592 *R* ^2^ = 0.7383 RMSE = 14.68 *p* < 2.200e‐16
k‐Nearest neighbors (k‐NN)	Training samples = 104, *k* = 8	MAE = 22.37 *r* = 0.3577 *R* ^2^ = 0.1279 RMSE = 31.14
Decision tree	Tree structure: Max depth = 6 Minimum sample split = 2 Minimum sample leaf = 1 Complexity parameter = 0.001 Variable importance: Undenatured protein = 51574.2 Surface Hydrophobicity = 22872.8 Zeta Potential = 12331.6	MAE = 11.92 *r* = 0.7608 *R* ^2^ = 0.5788 RMSE = 19.84
Gradient boosting machine (GBM)	Relative influence of variables: Undenatured protein = 40.89 Surface hydrophobicity = 35.68 Zeta potential = 23.43 Iterations = 100; Interaction depth = 7 Model shrinkage = 0.1 Minimum number of observations required in each terminal nodes = 5	MAE = 12.32 *r* = 0.8089 *R* ^2^ = 0.6543 RMSE = 16.83
Poisson Regression	Coefficients: Intercept = 4.232 (*p* < 2.000e‐16·***) Surface Hydrophobicity = −8.711e‐5 (*p* < 2.000e‐16·***) Zeta Potential = −1.465e‐2 (*p* = 4.750e‐09·***) Undenatured protein = 8.217e‐3 (*p* < 2.000e‐16·***) Dispersion parameter for Poisson family = 1 Null deviance: 2032.5 on 103 degrees of freedom Residual deviance: 933.6 on 100 degrees of freedom AIC: Inf Number of Fisher Scoring iterations: 5	MAE = 51.62 *r* = 0.6867 *R* ^2^ = 0.4715 RMSE = 58.85
Lasso Regression	Degree of freedom = 3% Deviance explained = 61.48 Lambda = 1, alpha = 1 Beta: Surface Hydrophobicity = −0.0038 Zeta Potential = −0.5527 Undenatured protein = 0.4602	MAE = 17.32 *r* = 0.6708 *R* ^2^ = 0.4499 RMSE = 22.31
Log‐linear regression	Coefficients: Intercept = 4.232 (*p* < 2.000e‐16·***) Surface hydrophobicity = −8.711e‐5 (*p* < 2.000e‐16·***) Zeta potential = −1.465e‐2 (*p* = 4.750e‐09·***) Undenatured protein = 8.217e‐3 (*p* < 2.000e‐16·***) Dispersion parameter for poisson family = 1 Null deviance: 2032.5 on 103 degrees of freedom Residual deviance: 933.6 on 100 degrees of freedom AIC: Inf Number of Fisher Scoring iterations: 5 Lambda = 1, alpha = 1	MAE = 20.71 *r* = 0.6286 *R* ^2^ = 0.3951 RMSE = 26.41

*Note:* Significance codes: 0 “***”; 0.01 “*”; 0.05 “.” 0.1.

Abbreviations: MAE, mean absolute error; *r*, correlation coefficient; *R* ^2^, coefficient of determination; RMSE, root mean squared error.

Based on conformation to physical constraints, maxima and minima for Sol were set at 0% and 100%, respectively (Figure S2). Linear regression over‐estimated the *Test* data for high Sol samples and predicted above 100% solubility. Regularization of the linear model using the Lasso model also resulted in Sol predictions above 100%. Log‐transformed linear regression or log‐linear model hugely over‐estimated the Sol for samples with high solubility, due to its exponential nature. Polynomial and spline regression models, which were used to capture non‐linearity in the dataset, over‐fit the data by predicting unexpected minima (< 0% Sol) values and maxima (> 100% Sol), thus hampering the reliability of the prediction model.

The rest of the trained models, namely, decision tree, random forest, gradient boosting machine, k‐nearest neighbors and *Gaussian* based Support Vector Regression models showed lower prediction errors within reasonable limits (< 20%; except for k‐nearest neighbors). The k‐nearest neighbors model over‐estimated the Sol for samples with 30%–50% solubility and under‐predicted at higher solubility (> 80%). These models, however, had good predictions for individual protein sources (Figure S2). For instance, the trained models predicted correctly the lowest Sol for hemp samples (rice protein was split in the *Test* data, hence not predicted in the results) and highest Sol in case of soy and chickpea proteins, following the trends of the *Train* and *Test* datasets as well as following the trends of box plot distributions (Figure S1a).

*Gaussian* based Support Vector Regression showed the highest prediction power within reasonable error limits ($R^{2}=0.8906;$ MAE = 7.4711; RMSE = 9.584), and prediction values between feasible minima and maxima for Sol, reflecting strong model performance. The *Gaussian* based Support Vector Regression model, which employs radial basis function kernel, has also been shown to have better prediction and accuracy compared to other classical models in other studies [43, 44, 45]. However, none of the previous studies were on protein related datasets. Our study illustrated, for the first time, the effectiveness of *Gaussian* based Support Vector Regression model for the prediction of plant protein Sol. Residual plots were used to illustrate differences between observed and predicted values plotted against the predicted variables to assess the model fitting and inadequacies. The residual plots of predicted Sol versus the residuals (differences between observed and predicted values) for *Gaussian* based Support Vector Regression model showed that the residuals were randomly distributed around the zero line (Figure S3a), with maximum deviation from the predicted Sol for 25%–75% Sol.

In addition to evaluating the performance of each model, done to understand the effect of individual variables/predictors ($X_{i}$) on the functionality of the proteins when other variables were kept constant, partial dependence plots analyses were performed. Figure S4 shows the partial dependence plots for predicted Sol against the normalized values of predictors, namely SH, ZP, and U for a few selected models (linear, polynomial, spline regression, random forest, SVM, *Gaussian* based Support Vector Regression, gradient boosting machine and decision tree models). Differences among the model plots were evident (Figure S4). Linear model fitted the data in straight lines, while polynomial and spline models highly over‐estimated Sol. Random forest, decision tree and gradient boosting machine models showed a discontinuous nature, and thus were not preferred [29]. “Pearson” correlation coefficients (r) and *p*‐values for the models fitted on individual predictors are given in Table S2. Regardless of the trained model types, SH was the dominant predictor of Sol (*p* < 0.05), followed by U (*p* < 0.05), similar to the trend observed in the experimental and scatterplots (Figure 1a,c). Predictions based on ZP alone highly under‐estimated Sol (even at *p* < 0.05), except for *Gaussian* based support vector regression model, thus requiring other variables in the models.

Presence of hydrophobic residues on the protein surface and the inherent SH govern how the protein molecules aggregate, with weakened protein‐water interactions negatively impacting protein functionality [46]. Although hydrophobic groups tend to be mostly buried in the interior moiety of the proteins, often hydrophobic residues are also present on the protein surfaces, depending on the protein source and/or the protein extraction/processing conditions [46]. Different proteins have distinct Sol relationships with the predictors used. Due to a good hydrophilic/hydrophobic balance and low SH values, soy protein samples exhibited the highest Sol. Negative correlation between SH and Sol of soy protein isolates was also reported previously [16, 18, 46]. At zero net (constant) charge, or ZP, the SH of soy and milk proteins was negatively correlated with Sol [16], in line with our observed results. Similar results hold for rice proteins as demonstrated by Paraman et al. [42]. Hemp protein, edestin (11S globulin), also imparts high SH leading to its lower Sol compared to other plant proteins [6, 7].

ZP also negatively affected Sol (Figure S4). Net surface charge on the protein surface governs the repulsive forces with surrounding protein molecules and stabilizes the protein dispersions. As the normalized value increased (0–1), ZP values increased in magnitude (becoming less negative), thus lowering the Sol. Similar observations were reported by Hayakawa and Nakai [16], as ZP became closer to 0 mV (at constant SH), the % insoluble protein increased. At ZP close to 0 mV, the protein molecules tend to aggregate and negatively impact the overall functionality. This observation is consistent with more recent findings by Jiang et al. [47], Yang et al. [48], and Gao et al. [49]. Hayakawa and Nakai [16] developed a polynomial regression model (degree = 2; *p* < 0.001) to quantify the effects of SH and ZP on soy protein solubility. However, they utilized a degree‐2 polynomial regression model for milk and soy proteins, unlike our present study, which explored other advanced ML models with better flexibility and prediction power.

The extent of denaturation and the level of undenatured protein, U (Equation 1) also affect protein solubility. Extent of denaturation is dependent on factors such as the protein extraction conditions that vary with protein sources. In this study, as an individual predictor, U was positively correlated with Sol (Figure S4), similar to the observations reported by Hayakawa and Nakai [16] for soy and milk proteins and in a recent study by Verfaillie et al. [50] for soy protein isolates. Distinctively, our study included U as a predictor of protein functional properties for more complex datasets obtained from diverse proteins with varying processing history. Such diverse datasets allowed for a wide applicability and generalized predictions. Overall, it was apparent that SH, ZP, and U were strong predictors of Sol for different types of plant proteins. Therefore, employing these predictors in the ML algorithms together effectively predicted the solubility of soy, pea, chickpea, camelina, rice, and hemp protein ingredients.

#### 3.2.2 Emulsifying Properties Prediction

**FIGURE 5:**
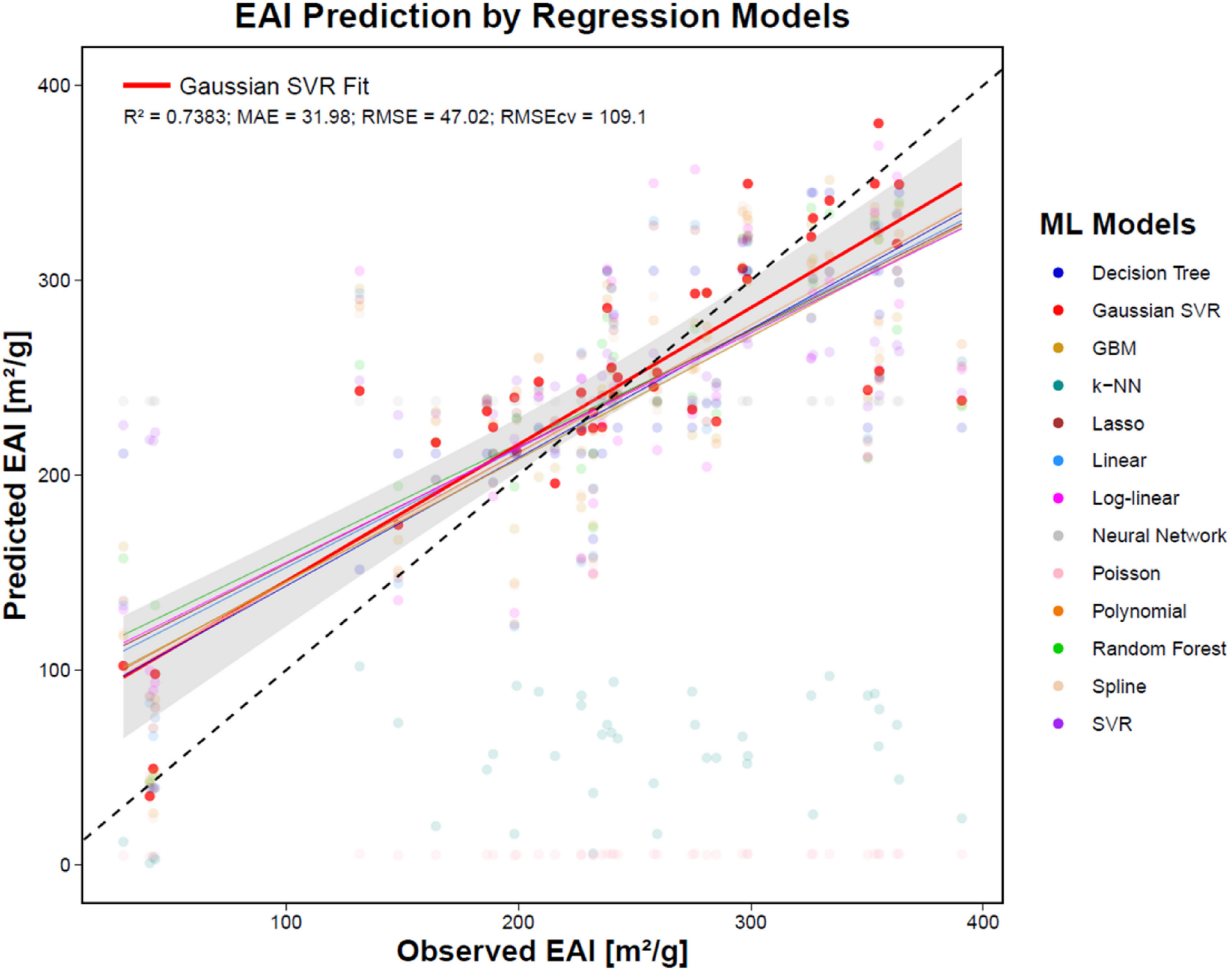
Actual versus predicted emulsifying activity index (EAI) plot based on fitting different machine learning (ML) models. Individual models are color coded as per legend. MAE, mean absolute error; RMSE, root mean squared error.

**FIGURE 6:**
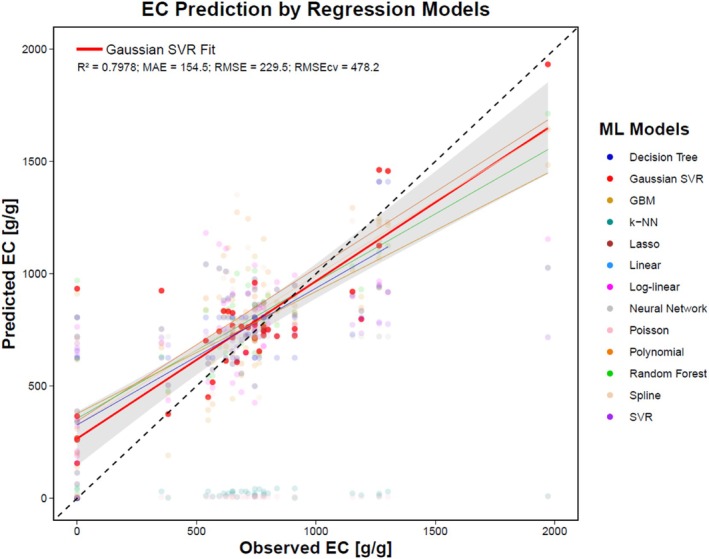
Actual versus predicted emulsifying capacity (EC) plot based on fitting different machine learning (ML) models. Individual models are color coded as per legend. MAE, mean absolute error; RMSE, root mean squared error.

The emulsifying properties of the different proteins were studied in terms of EAI [m^2^/g] and EC [g/g], although other parameters like emulsion stability, emulsion separation velocity, creaming rate can equally be considered to fall under the umbrella term—emulsifying properties. The actual ($Y_{i}$) versus predicted (${\hat{Y}}_{i}$) EAI and EC scatterplots generated using the trained ML prediction scatterplots are shown in Figures 5 and 6, respectively. The corresponding model parameters, significance values of coefficients, RMSE, MAE, and $R^{2}$ for the models with tuned hyperparameters are given in Tables 2 and 3. The trained models showed varied performance for predicting EAI and EC. The *Gaussian* based Support Vector Regression model showed the best fit for both EAI and EC with highest $R^{2}$ = 0.7383 and 0.7978, respectively, MAE of 31.98 and 154.5, and RMSE = 47.02 and 229.5, respectively. The prediction capability of the Poisson model for predicting EAI and EC was among the poorest (Figures 5 and 6, and Tables 2 and 3) ($R^{2}$ = 0.5810 and 0.3388, MAE = 237.6 and 746.2, RMSE = 254.5 and 893.7, for EAI and EC, respectively). The Poisson model under‐estimated all the *Test* data points. The k‐nearest neighbors, neural network, and support vector regression models also poorly predicted EAI and EC.

**TABLE 2:** Machine learning models comparison for protein emulsifying activity index: Model parameters and performance metrics.

Model	Model parameters	Performance metrics
Linear regression	Coefficients: Intercept = 241.7 (*p* = 5.420e‐14·***) Surface Hydrophobicity = −0.0078 (*p* = 2.320e‐6·***) Solubility = 1.764 (*p* = 7.320e‐10·***) Undenatured protein = −0.226 (*p* = 0.2660) Residual standard error: 52.4 on 100 degrees of freedom Multiple *R* ^2^ = 0.6843, Adjusted *R* ^2^ = 0.6749	MAE = 47.64 *r* = 0.7596 *R* ^2^ = 0.5770 RMSE = 59.68 *p* < 2.200e‐16
Polynomial regression	Coefficients: Intercept = 238.1 (*p* < 2.000e‐16·***) Surface hydrophobicity = −366.2 (*p* = 1.560e‐8·***) (Surface hydrophobicity)^2^ = −190.8 (*p* = 0.0008·***) (Surface hydrophobicity)^3^ = 37.85 (*p* = 0.4152) Solubility = 367.7 (*p* = 4.720e‐6·***) (Solubility)^2^ = −18.34 (*p* = 0.7660) (Solubility)^3^ = 16.88 (*p* = 0.7303) Undenatured protein = 93.78 (*p* = 0.2027) (Undenatured protein)^2^ = 184.4 (*p* = 0.0005·***) (Undenatured protein)^3^ = −100.9 (*p* = 0.0446·*) Residual standard error: 44.98 on 94 degrees of freedom Multiple *R* ^2^ = 0.7813, Adjusted *R* ^2^ = 0.7604	MAE = 41.44 *r* = 0.8051 *R* ^2^ = 0.6482 RMSE = 54.33 *p* < 2.200e‐16
Random forest	Tree structure: 500 trees % Variable explained = 74.42 Importance (Increase in Node Purity): Surface hydrophobicity = 302159.0 Solubility = 308019.8 Undenatured protein = 194940.1 Mean of squared residuals: 2139.0	MAE = 38.41 *r* = 0.7923 *R* ^2^ = 0.6277 RMSE = 56.11
Support vector machine regression (SVR)	Support vectors: SV type: Epsilon‐svr (regression) Number of support vectors = 8990 Epsilon = 0.1 cost C = 0.1 Kernel parameters Linear (vanilla) kernel function Objective function value = −6.252 Training error = 0.7523	MAE = 61.10 *r* = 0.6818 *R* ^2^ = 0.4649 RMSE = 81.61
Gaussian SVR	SV type: Epsilon‐svr (regression) Number of support vectors = 80 Epsilon = 0.1 cost C = 10 Gaussian radial basis kernel function Hyperparameter: sigma = 1 Objective function value = −159.4 Training error = 0.1221	MAE = 31.98 *r* = 0.8593 *R* ^2^ = 0.7383 RMSE = 47.02 RMSE_CV_ = 109.1
Neural network	Network architecture: Hidden layers: 7, 5 Activation function: Logistic: $f\left(x\right)=\frac{1}{1+e^{-x}}$	MAE = 69.84 *r* = _ *R* ^2^ = _ RMSE = 91.56
Spline regression	Coefficients: Intercept = 167.0 (*p* = 6.410e‐6·***) Surface hydrophobicity = −8.381 (*p* = 0.7455) (Surface hydrophobicity)^2^ = −39.32 (*p* = 0.4357) (Surface hydrophobicity)^3^ = −154.3 (*p* = 2.120e‐6·***) Solubility = 74.85 (*p* = 0.0150·*) (Solubility)^2^ = 59.79 (*p* = 0.1101) (Solubility)^3^ = 112.6 (*p* = 0.0029·**) (Solubility)^4^ = 152.0 (*p* = 0.0122·*) (Solubility)^5^ = 111.2 (*p* = 7.190e‐5·***) Undenatured protein = −6.922 (*p* = 0.8527) (Undenatured protein)^2^ = −12.72 (*p* = 0.7260) (Undenatured protein)^3^ = 84.61 (*p* = 0.0120·*) Residual standard error: 43.70 on 94 degrees of freedom Multiple *R* ^2^ = 0.7980, Adjusted *R* ^2^ = 0.7738	MAE = 41.03 *r* = 0.8119 *R* ^2^ = 0.6593 RMSE = 53.50 *p* < 2.200e‐16
k‐Nearest neighbors (k‐NN)	Training samples = 104, k = 10	MAE = 186.4 *r* = 0.4266 *R* ^2^ = 0.1820 RMSE = 204.1
Decision tree	Tree structure: Non‐leaf nodes = 7 Variable importance: Undenatured protein = 139337.0 Surface Hydrophobicity = 554636.3 Solubility = 504546.0	MAE = 37.42 *r* = 0.8041 *R* ^2^ = 0.6466 RMSE = 54.66
Gradient boosting machine (GBM)	Relative influence of variables: Undenatured protein = 10.61 Surface hydrophobicity = 43.41 Solubility = 45.98 Iterations = 50; Interaction depth = 7 Model shrinkage = 0.1 Minimum number of observations required in each terminal nodes = 5	MAE = 37.81 *r* = 0.7803 *R* ^2^ = 0.6088 RMSE = 57.75
Poisson regression	Coefficients: Intercept = 5.556 (*p* < 2e‐16·***) Surface hydrophobicity = −4.453e‐5 (*p* < 2.000e‐16·***) Solubility = 8.247e‐3 (*p* < 2.000e‐16·***) Undenatured protein = −2.166e‐3 (*p* < 2.000e‐16·***) Dispersion parameter for poisson family = 1 Null deviance: 4518 on 103 degrees of freedom Residual deviance: 1762 on 100 degrees of freedom AIC: Inf Number of Fisher Scoring iterations: 4	MAE = 237.6 *r* = 0.7623 *R* ^2^ = 0.5810 RMSE = 254.5
Lasso regression	Degree of freedom = 3% Deviance explained = 68.39 Lambda = 1 Beta: Surface hydrophobicity = −0.0078 Solubility = 1.6967 Undenatured protein = −0.1601	MAE = 47.99 *r* = 0.7575 *R* ^2^ = 0.5739 RMSE = 59.82
Log‐linear regression	Coefficients: Intercept = 5.556 (*p* < 2.000e‐16·***) Surface hydrophobicity = −4.453e‐5 (*p* < 2.000e‐16·***) Solubility = 8.247e‐3 (*p* < 2.000e‐16·***) Undenatured protein = −2.166e‐3 (*p* < 2.000e‐16·***) Dispersion parameter for Poisson family = 1 Null deviance: 4518 on 103 degrees of freedom Residual deviance: 1762 on 100 degrees of freedom AIC: Inf Number of Fisher Scoring iterations: 4	MAE = 52.50 *r* = 0.7125 *R* ^2^ = 0.5077 RMSE = 65.09

*Note:* Significance codes: 0 “***”; 0.001 “**”; 0.01 “*”; 0.05 “.” 0.1.

Abbreviations: MAE, mean absolute error; *r*, correlation coefficient; *R* ^2^, coefficient of determination; RMSE, root mean squared error.

**TABLE 3:** Machine learning models comparison for protein emulsifying capacity: Model parameters and performance metrics.

Model	Model parameters	Performance metrics
Linear regression	Coefficients: Intercept = 1.461e+3 (*p* = 9.060e‐9·***) Surface Hydrophobicity = −6.270e‐2 (*p* = 2.720e‐14·***) Zeta Potential = −2.171 (*p* = 0.7355) Undenatured protein = −2.078 (*p* = 0.0566.) Residual standard error: 313 on 98 degrees of freedom Multiple *R* ^2^ = 0.4521, Adjusted *R* ^2^ = 0.4354	MAE = 266.7 *r* = 0.5816 *R* ^2^ = 0.3382 RMSE = 403.5 *p* = 8.492e‐13
Polynomial regression	Coefficients: Intercept = 720.6 (*p* < 2.000e‐16·***) Surface Hydrophobicity = −2247 (*p* = 4.470e‐11·***) (Surface Hydrophobicity)^2^ = −195.5 (*p* = 0.5170) (Surface Hydrophobicity)^3^ = 133.8 (*p* = 0.6218) (Surface Hydrophobicity)^4^ = −79.50 (*p* = 0.7679) Zeta Potential = −212.5 (*p* = 0.4331) (Zeta Potential)^2^ = 1623 (*p* = 3.400e‐8·***) (Zeta Potential)^3^ = 888.4 (*p* = 0.0012·**) (Zeta Potential)^4^ = −100.3 (*p* = 0.7131) Undenatured protein = −216.2 (*p* = 0.4744) (Undenatured protein)^2^ = 343.9 (*p* = 0.2438) (Undenatured protein)^3^ = −741.7 (*p* = 0.0089·**) (Undenatured protein)^4^ = −48.37 (*p* = 0.8648) Residual standard error: 255.1 on 89 degrees of freedom Multiple *R* ^2^ = 0.6694, Adjusted *R* ^2^ = 0.6248	MAE = 225.5 *r* = 0.8011 *R* ^2^ = 0.6417 RMSE = 306.6 *p* < 2.200e‐16
Random forest	Tree structure: 500 trees % Variable explained = 67.39 Importance (Increase in Node Purity): Surface Hydrophobicity = 6 776 464 Zeta Potential = 6 063 689 Undenatured protein = 3 279 894 Mean of squared residuals: 56183.8	MAE = 181.0 *r* = 0.8772 *R* ^2^ = 0.7694 RMSE = 264.0
Support vector machine regression (SVR)	Support vectors: SV type: Epsilon‐svr (regression) Number of Support Vectors = 83 Epsilon = 0.1 cost C = 0.1 Gamma = 0.01 Kernel parameters Linear (vanilla) kernel function Objective function value = −5.685	MAE = 307.8 *r* = 0.4265 *R* ^2^ = 0.1819 RMSE = 481.8
Gaussian SVR	SV type: Epsilon‐svr (regression) Number of support vectors = 78 Epsilon = 0.1 cost C = 1 Gamma = 0.01 Gaussian Radial Basis kernel function Hyperparameter: sigma = 0.01 Objective function value = −235.6 Training error = 0.1669	MAE = 154.5 *r* = 0.8932 *R* ^2^ = 0.7978 RMSE = 229.5 RMSE_CV_=
Neural network	Network architecture: Hidden layers: 7, 5 Activation function: Logistic: $f\left(x\right)=\frac{1}{1+e^{-x}}$	MAE = 321.9 *r* = _ *R* ^2^ = _ RMSE = 495.5
Spline regression	Coefficients: Intercept = 1571 (*p* = 3.970e‐8·***) Surface Hydrophobicity = −124.7 (*p* = 0.4300) (Surface Hydrophobicity)^2^ = −247.1 (*p* = 0.2560) (Surface Hydrophobicity)^3^ = −401.8 (*p* = 0.0398·*) (Surface Hydrophobicity)^4^ = −752.3 (*p* = 0.0553.) (Surface Hydrophobicity)^5^ = −899.3 (*p* = 1.090e‐6·***) Zeta Potential = −572.8 (*p* = 0.0034·**) (Zeta Potential)^2^ = −680.9 (*p* = 0.0003·***) (Zeta Potential)^3^ = −695.7 (*p* = 0.1674) (Zeta Potential)^4^ = 518.6 (*p* = 0.0050·**) Undenatured protein = 1.890 (*p* = 0.9944) (Undenatured protein)^2^ = −234.2 (*p* = 0.2147) (Undenatured protein)^3^ = 262.8 (*p* = 0.2450) (Undenatured protein)^4^ = −530.8 (*p* = 0.1523) (Undenatured protein)^5^ = −103.9 (*p* = 0.6721) Residual standard error: 254.3 on 87 degrees of freedom Multiple *R* ^2^ = 0.6789, Adjusted *R* ^2^ = 0.6272	MAE = 216.4 *r* = 0.8212 *R* ^2^ = 0.6744 RMSE = 288.6 *p* = 5.388e‐16
k‐Nearest Neighbors (k‐NN)	Training samples = 104, *k* = 9	MAE = 734.4 *r* = 0.0665 *R* ^2^ = 0.0044 RMSE = 883.3
Decision Tree	Tree structure: Max depth = 4 Minimum sample split = 2 Minimum sample leaf = 1 Complexity parameter = 0.01 Variable importance: Undenatured protein = 1 508 028 Surface Hydrophobicity = 8 721 273 Zeta Potential = 6 000 991	MAE = 160.8 *r* = 0.8849 *R* ^2^ = 0.7830 RMSE = 238.8
Gradient Boosting Machine (GBM)	Relative Influence of variables: Undenatured protein = 12.39 Surface Hydrophobicity = 56.15 Zeta Potential = 31.49 Iterations = 50; Interaction depth = 7 Model shrinkage = 0.1 Minimum number of observations required in each terminal nodes = 5	MAE = 210.1 *r* = 0.7816 *R* ^2^ = 0.6109 RMSE = 314.0
Poisson Regression	Coefficients: Intercept = 7.885 (*p* < 2.000e‐16·***) Surface Hydrophobicity = −1.161e‐4 (*p* < 2.000e‐16·***) Zeta Potential = −2.735e‐3 (*p* = 9.340e‐5·***) Undenatured protein = −3.990e‐3 (*p* < 2.000e‐16·***) Dispersion parameter for Poisson family = 1 Null deviance: 31012 on 101 degrees of freedom Residual deviance: 17852 on 98 degrees of freedom AIC: Inf Number of Fisher Scoring iterations: 5	MAE = 746.2 *r* = 0.5820 *R* ^2^ = 0.3388 RMSE = 893.7
Lasso regression	Degree of freedom = 3% Deviance explained = 45.21 Lambda = 1, alpha = 1 Beta: Surface Hydrophobicity = −0.0624 Zeta Potential = −2.033 Undenatured protein = −2.042	MAE = 266.2 *r* = 0.5835 *R* ^2^ = 0.3405 RMSE = 403.0
Log‐linear Regression	Coefficients: Intercept = 7.885 (*p* < 2.000e‐16·***) Surface Hydrophobicity = −1.161e‐4 (*p* < 2.000e‐16·***) Zeta Potential = −2.735e‐3 (*p* = 9.340e‐5·***) Undenatured protein = −3.990e‐3 (*p* < 2.000e‐16·***) Dispersion parameter for Poisson family = 1 Null deviance: 31012 on 101 degrees of freedom Residual deviance: 17852 on 98 degrees of freedom AIC: Inf Number of Fisher Scoring iterations: 5 Lambda = 1, alpha = 1	MAE = 288.4 *r* = 0.5505 *R* ^2^ = 0.3031 RMSE = 413.1

*Note:* Significance codes: 0 “***”; 0.001 “**”; 0.01 “*”; 0.05 “.” 0.1.

Abbreviations: MAE, mean absolute error; *r*, correlation coefficient; *R* ^2^, coefficient of determination; RMSE, root mean squared error.

To adhere to physical constraints for EAI and EC, the minima was set at 0, meaning only non‐negative values were accepted and models predicting negative values were not preferred (Figures S5 and S6). Linear regression under‐estimated the *Test* data for high EAI and EC samples, and the performance was not improved by regularization using Lasso model, which poorly predicted the emulsifying properties above 1500 m^2^/g EAI and 300 g/g EC. These models also predicted negative values for samples with low EC. Log‐transformed linear regression did not show any negative values, but it under‐estimated the emulsifying properties and performed poorer than the linear model (Figure S6).

Polynomial and spline regression better predicted EAI and EC then linear model, albeit with the prediction of unexpected minima (< 0 m^2^/g or g/g for EAI or EC) values. The remining trained models, viz. decision tree, random forest, gradient boosting machine, and *Gaussian* based Support Vector Regression models showed low prediction errors within reasonable limits (<15%). The random forest and gradient boosting machine model over‐estimated the EAI and EC for samples with <200 m^2^/g EAI and < 500 g/g EC and under‐predicted at higher values for the *Test* data. The latter also predicted negative values for EC (Figure S6).

These trained models had relatively good predictions for individual protein sources, with the exception of rice protein (Figures S5 and S6). For all the trained models, the predicted EAI was the lowest for hemp samples, followed by rice samples. The highest EAI was predicted for soy samples, similar to the trends of the *Train* and *Test* data scatterplots as well as the box plot distributions (Figures 1d–g and S1b). Contrary for EC, lowest values were predicted for hemp, as opposed to rice in the dataset, probably due to lower datapoints for the latter. Highest EC was predicted by all the models for camelina samples, except for the linear, regularized and log‐transformed models (Figure S6).

Overall, the *Gaussian* based Support Vector Regression showed the highest prediction capability within reasonable error limits and prediction values for EAI and EC between feasible minima and maxima. The residual plots of predicted EAI and EC versus the residual values (Figure S3b,c) for *Gaussian* based Support Vector Regression model showed that the residuals were randomly distributed around the zero line, though maximum deviations for 100–300 m^2^/g EAI and > 800 g/g EC were observed. This observation indicated that the model did not perform well in that range. Nevertheless, a random distribution around the zero line suggested no systematic biases in the model, accounting effectively for the underlying patterns in the data.

Partial dependence of variables was plotted (Figures S7 and S8) to show the effect of each individual predictor ($X_{i}$) on the emulsifying properties. The plots show the partial dependence of predicted EAI and EC against the normalized values of predictors, SH, ZP and U for the linear, polynomial, spline regression, random forest, support vector regression, *Gaussian* based Support Vector Regression, gradient boosting machine and decision tree models. “Pearson” correlation coefficients (r) and *p*‐values for the models fitted on individual predictors are given in Table S2.

The polynomial and spline models tend to over‐fit the data for all the modeled predictors [29]. Random forest, and decision tree models showed a discontinuity for both EAI and EC posing challenges in predictions. The normalized SH was the single most dominant predictor of EAI and EC (*p* < 0.05) (Figures S7 and S8), following the observed experimental data trend and scatterplots (Figure 1d,h), explaining most of the variations in the dataset. However, prediction of emulsifying properties using the U predictor, highly under‐estimated EAI and EC. The effect was not significant (*p* > 0.05) for the prediction of EC using polynomial, spline, random forest, decision tree, *Gaussian* based Support Vector Regression, and gradient boosting machine models. This observation necessitated the use of other predictors in the models such as Sol and ZP. Emulsifying properties of proteins are governed by how readily they are available at the oil/water interface. EAI gives an indication of the interfacial area stabilized by a unit weight of protein in terms of the turbidity of a dilute emulsion [8, 51]. On the other hand, EC represents the amount of oil that can be emulsified per unit weight of protein under specific conditions [8, 51].

The protein surface properties play an important role in its emulsification behavior. A good balance of hydrophilic and hydrophobic regions on the surface is necessary for good emulsifying properties. Therefore, SH could greatly impact the emulsifying properties of proteins and could contribute to enhanced prediction accuracy of models, as evidenced by previous research [8, 24, 52, 53]. The SH negatively correlated with emulsifying properties (r = −0.693 with EAI and r = −0.651 with EC; *p* < 0.05), contrary to previous observations for soy protein isolates [53, 54], rapeseed and sunflower proteins [24, 53], pea protein isolates [24], chickpea, fava bean and legume protein isolates [8], where SH positively correlated with emulsification parameters. This contradictory observation could be attributed to the magnitude of the measured SH. Very high H, as is the case for hemp and rice protein, will most likely lead to hydrophobic interactions and self‐association impairing the balanced interactions at the oil–water interface, thus decreasing protein adsorption [54].

Protein denaturation state also affects the emulsification process. Protein denaturation led to unfolding of the molecules, thus exposing hydrophobic residues and modulating the protein surface properties [24, 55]. Although U, as an individual predictor, weakly correlated with EAI and EC (Figures 1f,j, S7 and S8), it was included in the trained ML models for predicting EAI and EC, due to its potential indirect impact on the interactions at the interface.

Sol showed a strong positive correlation (r = 0.744; *p* < 0.05) with the interfacial activity of proteins. Sol is an important prerequisite for emulsifying activity, since proteins need to be soluble in the aqueous dispersion. Insoluble proteins can precipitate at the interface leading to emulsion destabilization [42]. Soluble, flexible proteins provide stabilization of a relatively high surface area [54]. Almost similar box plot distribution for Sol and EAI was observed across individual protein sources (Figure S1a,b). Therefore, Sol along with SH was used in the trained ML models for EAI prediction. Previous research also showed that both solubility and surface hydrophobicity have an effort on EAI of plant, milk, and meat proteins [24, 52] since they both affect the EAI. Sol was a better predictor for EAI for samples with lower solubility (<50%), whereas for higher solubility (>50%), SH influenced EAI prediction. While earlier studies [24, 52] considered only Sol and SH for modeling, our study incorporated the U predictor as well to improve the prediction of EAI.

For EC prediction, ZP was considered (in addition to SH and U) as it negatively yet weakly correlated with EC (Figure S8). This observation indicated that low surface charge (where net charge approaches 0 mV), negatively affected EC by destabilizing emulsions as demonstrated previously [8, 52, 56].

Expectedly, rice and hemp proteins showed the lowest EC, possibly due to their highest SH and lowest Sol among the samples (Figure S1c). The higher EC of camelina protein compared to soy and pea protein, could be attributed to the smaller size of its 11S globulin (cruciferin) and its higher surface hydrophobicity and molecular flexibility compared to soy and pea 11S globulins (glycinin and legumin, respectively). These molecular differences could have allowed the camelina protein to readily migrate to the interface [9, 57]. Overall, the predictors SH, ZP, Sol, and U adequately predicted EAI and EC for different types of plant proteins when used in the *Gaussian* based Support Vector Regression model.

#### 3.2.3 Gel Strength Prediction

**FIGURE 7:**
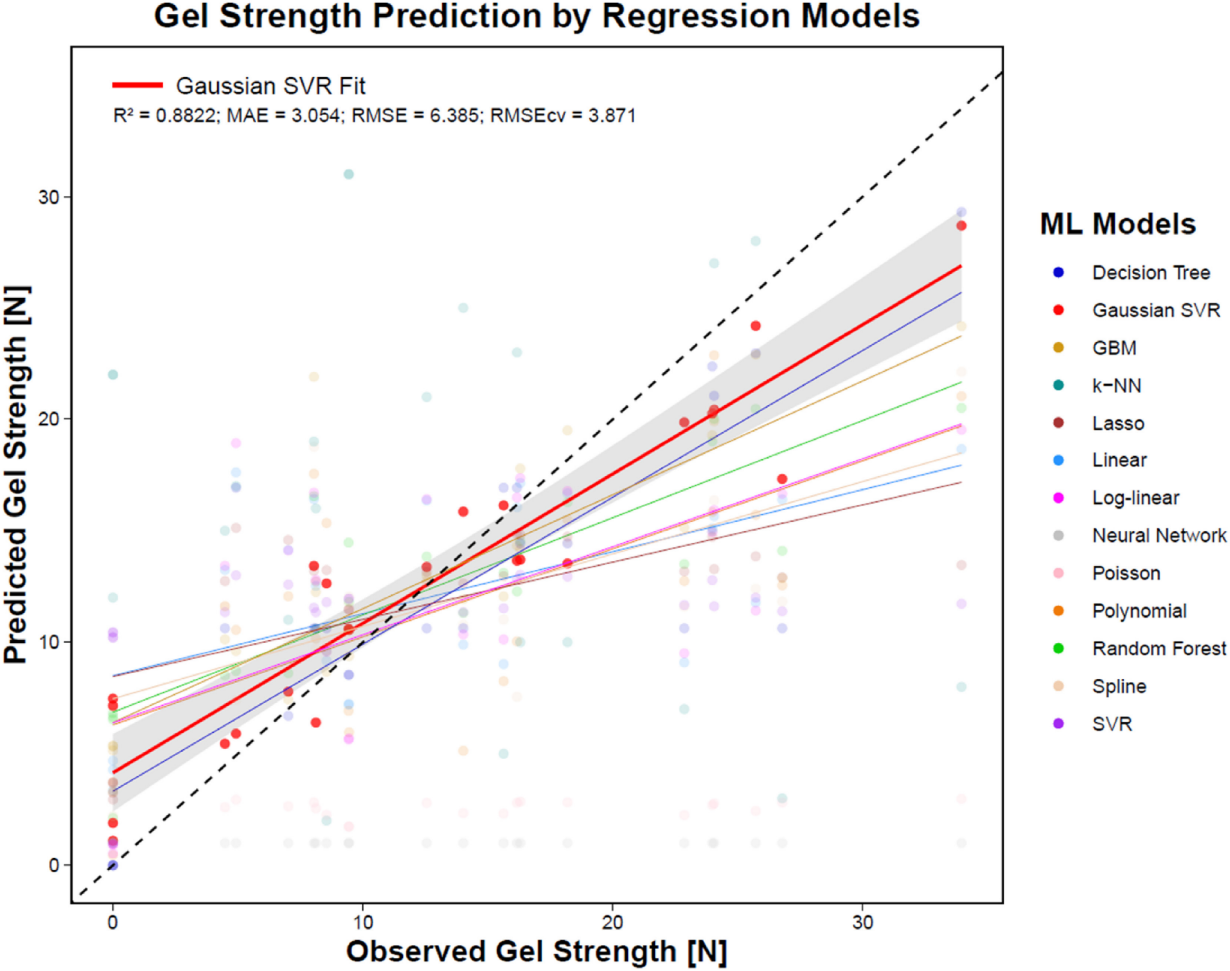
Actual versus predicted gel strength (Gel) plot based on fitting different machine learning (ML) models. Individual models are color coded as per legend. MAE, mean absolute error; RMSE, root mean squared error.

The actual ($Y_{i}$) versus predicted (${\hat{Y}}_{i}$) Gel scatterplot using the different ML algorithms is shown in Figure 7. The associated model parameters, coefficients, and their statistical significance, along with the RMSE, MAE and $R^{2}$ for the fitted models with tuned hyperparameters are given in Table 4. The predictions of Gel using the evaluated ML algorithms for the different protein samples, namely, soy, pea, chickpea, and pennycress, showed varied performance. The poorest performing models were the neural network, k‐nearest neighbor, and Poisson models, in terms of very high prediction errors (Table 4). With very high prediction errors (up to 60% of max Gel values), these models under‐estimated Gel with a mean prediction value of 1.000, 15.89, and 2.109 N for neural network, k‐nearest neighbors, and Poisson models, respectively. Similarly, the support vector regression model performed poorly with a mean prediction for Gel of 55.03 N, over‐fitting at lower gel strength values (< 10 N) and under‐fitting at higher values (> 20 N). Hence, these models did not adequately predict Gel of the evaluated plant proteins. The *Gaussian* based Support Vector Regression model gave the best fit for Gel, in terms of lowest MAE (3.054) and RMSE (6.385), and highest $R^{2}$ (0.8822) values. This finding indicated that Gaussian SVR model explained above 88% of the variance in the experimental data, with an prediction error < 10 units, highlighting good accuracy within reasonable errors.

**TABLE 4:** Machine learning models comparison for protein gel strength: Model parameters and performance metrics.

Model	Model parameters	Performance metrics
Linear regression	Coefficients: Intercept = 0.5761 (*p* = 0.9100) Solubility = 0.1042 (*p* = 0.2175) Water holding capacity = 0.1338 (*p* = 0.00354·**) *β*‐sheet content = −0.4698 (*p* = 0.05480.) Undenatured protein = 0.0624 (*p* = 0.3744) Polymerized protein = −0.2101 (*p* = 0.0414·*) Residual standard error: 8.029 on 62 degrees of freedom Multiple *R* ^2^ = 0.2616, Adjusted *R* ^2^ = 0.2020	MAE = 6.287 *r* = 0.5982 *R* ^2^ = 0.3578 RMSE = 7.671 *p* = 0.0017
Polynomial regression	Coefficients: Intercept = 13.07 (*p* < 2.000e‐16·***) Solubility = 25.23 (*p* = 0.2417) (Solubility)^2^ = −17.87 (*p* = 0.1175) Water holding capacity = 21.34 (*p* = 0.0161·*) (Water holding capacity)^2^ = −19.57 (*p* = 0.2049) *β*‐sheet content = −28.21 (*p* = 0.0551.) (*β*‐sheet content)^2^ = −26.25 (*p* = 0.0553.) Undenatured protein = 12.60 (*p* = 0.5433) (Undenatured protein)^2^ = −9.364 (*p* = 0.3404) Polymerized protein = −13.79 (*p* = 0.1868) (Polymerized protein)^2^ = 14.18 (*p* = 0.0961.) Residual standard error: 7.610 on 57 degrees of freedom Multiple *R* ^2^ = 0.3645, Adjusted *R* ^2^ = 0.2830	MAE = 5.642 *r* = 0.7275 *R* ^2^ = 0.5292 RMSE = 6.857 *p* = 0.0008
Random forest	Tree structure: 500 trees % Variable explained = 39.06 Importance (Increase in Node Purity): Solubility = 1134.8 Water holding capacity = 1072.1 *β*‐sheet content = 999.2 Undenatured protein = 860.7 Polymerized protein = 1029.5 Mean of squared residuals: 48.50	MAE = 4.784 *r* = 0.8660 *R* ^2^ = 0.7499 RMSE = 5.833
Support vector machine regression (SVR)	Support vectors: SV type: Epsilon‐svr (regression) Number of Support Vectors = 66 Epsilon = 0.1 cost C = 0.1 Gamma = 0.01 Kernel parameters Linear (vanilla) kernel function Objective function value = −4.268 Training error = 0.9396	MAE = 7.610 *r* = 0.4431 *R* ^2^ = 0.1963 RMSE = 9.161
Gaussian SVR	SV type: Epsilon‐svr (regression) Number of Support Vectors = 46 Epsilon = 0.1 cost C = 10 Gamma = 1 Gaussian Radial Basis kernel function Hyperparameter: sigma = 1 Objective function value = −65.63 Training error = 0.0401	MAE = 3.054 *r* = 0.9393 *R* ^2^ = 0.8822 RMSE = 6.385 RMSE_CV_ = 3.871
Neural network	Network architecture: Hidden layers: 7, 5 Activation function: Logistic: $f\left(x\right)=\frac{1}{1+e^{-x}}$	MAE = 12.26 *r* = _ *R* ^2^ = _ RMSE = 15.19
Spline regression	Coefficients: Intercept = 1.577 (*p* = 0.9117) Solubility = 7.685 (*p* = 0.4556) (Solubility)^2^ = −1.987 (*p* = 0.8757) (Solubility)^3^ = −4.971 (*p* = 0.8393) (Solubility)^4^ = 8.561 (*p* = 0.2797) Water holding capacity = 18.54 (*p* = 0.0490·*) (Water holding capacity)^2^ = 6.041 (*p* = 0.7970) (Water holding capacity)^3^ = 20.08 (*p* = 0.1455) *β*‐sheet content = −11.41 (*p* = 0.1753) (*β*‐sheet content)^2^ = 19.45 (*p* = 0.0167·*) (*β*‐sheet content)^3^ = −14.75 (*p* = 0.0613.) (*β*‐sheet content)^4^ = 10.47 (*p* = 0.4332) (*β*‐sheet content)^5^ = 1.835 (*p* = 0.8702) Undenatured protein = 2.143 (*p* = 0.8184) (Undenatured protein)^2^ = −3.388 (*p* = 0.7345) (Undenatured protein)^3^ = −4.915 (*p* = 0.5861) Polymerized protein = −3.597 (*p* = 0.5838) (Polymerized protein)^2^ = −14.19 (*p* = 0.2703) (Polymerized protein)^3^ = −3.058 (*p* = 0.4119) Residual standard error: 7.302 on 49 degrees of freedom Multiple *R* ^2^ = 0.5172, Adjusted *R* ^2^ = 0.3399	MAE = 5.120 *r* = 0.7539 *R* ^2^ = 0.5683 RMSE = 6.703 *p* = 0.0015
k‐Nearest neighbors (k‐NN)	Training samples = 104, *k* = 1	MAE = 11.80 *r* = −0.0797 *R* ^2^ = 0.0064 RMSE = 13.82
Decision tree	Tree structure: Max depth = 6 Minimum sample split = 2 Minimum sample leaf = 1 Complexity parameter = 0.001 Variable importance: Solubility = 1666 Water holding capacity = 2171.5 *β*‐sheet content = 2765.9 Undenatured protein = 1926 Polymerized protein = 1012	MAE = 3.367 *r* = 0.8320 *R* ^2^ = 0.6922 RMSE = 5.350
Gradient boosting machine (GBM)	Relative Influence of variables: Solubility = 19.98 Water holding capacity = 23.58 *β*‐sheet content = 27.10 Undenatured protein = 9.353 Polymerized protein = 20.10 Iterations = 100; Interaction depth = 3 Model shrinkage = 0.1 Minimum number of observations required in each terminal nodes = 5	MAE = 4.291 *r* = 0.8206 *R* ^2^ = 0.6734 RMSE = 5.697
Poisson regression	Coefficients: Intercept = −0.2656 (*p* = 0.5871) Solubility = 0.0069 (*p* = 0.0395·*) Water holding capacity = 0.0293 (*p* = 6.260e‐9·***) *β*‐sheet content = −0.03057 (*p* = 0.0016·**) Undenatured protein = 0.0047 (*p* = 0.0522.) Polymerized protein = −0.0163 (*p* = 4.590e‐6·***) Dispersion parameter for Poisson family = 1 Null deviance: 428.8 on 67 degrees of freedom Residual deviance: 277.5 on 62 degrees of freedom AIC: Inf Number of Fisher Scoring iterations: 5	MAE = 10.82 *r* = 0.6475 *R* ^2^ = 0.4193 RMSE = 13.92
Lasso regression	Degree of freedom = 3% Deviance explained = 17.33 Lambda = 1, alpha = 1 Beta: Solubility = 0.0240 Water holding capacity = 0.0983 *β*‐sheet content = _ Undenatured protein = 0.0082 Polymerized protein = _	MAE = 6.039 *r* = 0.6246 *R* ^2^ = 0.3901 RMSE = 7.712
Log‐linear regression	Coefficients: Intercept = −0.2656 (*p* = 0.5871) Solubility = 0.0069 (*p* = 0.0395·*) Water holding capacity = 0.0293 (*p* = 6.260e‐9·***) *β*‐sheet content = −0.0306 (*p* = 0.0016·**) Undenatured protein = 0.0047 (*p* = 0.0522.) Polymerized protein = −0.0163 (*p* = 4.590e‐6·***) Dispersion parameter for Poisson family = 1 Null deviance: 428.8 on 67 degrees of freedom Residual deviance: 277.5 on 62 degrees of freedom AIC: Inf Number of Fisher Scoring iterations: 5 Lambda = 1, alpha = 1	MAE = 5.856 *r* = 0.6207 *R* ^2^ = 0.3853 RMSE = 7.524

Model selection based on conformation to physical constraints included setting minima for Gel at 0 N, without any maxima values (Figure S9). Linear regression under‐estimated the *Test* data at high Gel values and also predicted negative Gel values. Linear regression model predicting unrealistic minima (negative values for gel strength) has also been demonstrated by Lie‐Piang et al. [29]. Improvement of linear model by penalty terms using regularization techniques employing Lasso method did not further enhance predictability and under‐estimated Gel for samples with high gel strength. Log‐transformed linear regression, on the other hand, slightly improved the Gel predictions, owing to its exponential behavior, as was similarly observed by Lie‐Piang et al. [29]. Polynomial and spline regression models over‐fitted the data, and predicted unrealistic minima (< 0 N for Gel) values. On the other hand, the remaining models, viz., decision tree, random forest, gradient boosting machine, and *Gaussian* based Support Vector Regression models showed much lower prediction errors under reasonable limits (<25%) compared to the other models. However, these models under‐estimated the Gel for the *Test* data at high gel strength (>25 N) (Figure 7). In addition, these models did not have accurate predictions for individual protein sources (Figure S9).

Chickpea samples had the highest mean Gel (16.23 N), followed by soy (13.76 N), pea (12.70 N), and pennycress (8.412 N) (Figures 2 and S1d). However, the trained models, gradient boosting machine and decision tree, inaccurately predicted the lowest Gel values for chickpea and pea samples and the highest Gel values in case of soy and pea (Figure S9). The observed trends were different for the *Train* and *Test* datasets and associated box plot distributions (Figure S1d). The discrepancies may have emerged due to poor splitting and the smaller number of datapoints for *Test* data (*n* = 24) compared to *Train* data (*n* = 68), leading to prediction errors for individual proteins. Nevertheless, these trained models gave prediction values above the feasible minima of Gel (≥ 0 N; non‐negative values), with the *Gaussian* based Support Vector Regression showing highest prediction power within reasonable error limits. The *Gaussian* based Support Vector Regression model outperformed all the other trained models for the prediction of Gel, similar to the case of the other evaluated functional properties. The residual plots of predicted Gel versus the model residuals (Figure S3d) for *Gaussian* based Support Vector Regression model showed that the residuals were randomly distributed around the zero line, with maximum deviation from the predicted values at 8–15 N Gel.

Mathematical modeling of food protein gelation has also been carried out by other researchers. Gupta et al. [58] used the neural networks model or in silico classification of the gelling ability of peptides. Lie‐Piang et al. [29] predicted gel strength (in terms of Young's modulus [kPa]) for yellow pea ingredients, where neural networks showed the most accurate prediction, contrary to our findings. The researchers did not test *Gaussian* based Support Vector Regression model; therefore, a direct comparison is not possible. In addition, Lie‐Piang et al. [29] used ingredient composition (w/w %) such as starch, protein, fibers, instead of protein structural characteristics, for the development of prediction models. The choice of input variables ($Y_{i}$) would most likely affect the performance of prediction models. Composition‐driven modeling of gel strength is more sensitive to initial conditions, since 10%–15% protein (w/w) is needed for most globular plant proteins to yield a strong gel, compared to linear polysaccharides (starch systems) (< 1%, w/w) [17, 59]. Gel strength (g) of industrial gelatin was predicted accurately using partial least square regression of near‐infrared spectroscopy data [60]. Smith et al. [61] used multiple linear regression employing temperature–time history, protein concentration, and pH for prediction of the gel strength (as viscosity index) of heat‐induced chicken myofibrillar protein gels. In comparison to other studies, and for the first time, Gel for the tested proteins was accurately predicted when *Gaussian* based Support Vector Regression model was employed, using only macromolecular protein structural characteristics, highlighting the strength of our study.

Partial dependence plots to visualize the effects of individual predictors ($X_{i}$), on Gel, while keeping other predictors constant, is shown in Figure S10. The predicted Gel for normalized values of predictors, namely Sol, WHC, βs, U, and SP, varied to some extent among few selected models (linear, polynomial, spline regression, log‐linear, random forest, SVM regression, *Gaussian* based Support Vector Regression, and decision tree models). All the models under‐estimated Gel when fitted on the trained dataset. Linear and support vector regression models fitted the data linearly, while polynomial models over‐fitted the data. The log‐linear model resulted in continuous non‐negative curve with an exponential fit, as expected. Spline models predicted unrealistic negative values. Random forest and decision tree models showed discontinuous curves and thus performed inadequately as evident in the partial dependence plots (Figure S10). This observation confirmed that these models failed to fully capture the complex relations between independent variables and Gel. Some of these observations are in line with those of Lie‐Piang et al. [29].

Regardless of the trained model types, it was evident that U was the dominant predictor of Gel (*p* < 0.05), followed by WHC (*p* < 0.05) (Table S2), similar to the trend observed in the experimental data scatterplots (Figure 2c,e). However, the contribution of WHC alone for bringing a unit change in Gel was low (Figure S10), contrary to SP and βs, which had the highest effect on Gel (even though the relation was not significant). Predictions based on Sol alone highly under‐estimated Gel (*p* < 0.05). However, the intricate relation among these variables (Sol, WHC, U, SP, βs) was further evaluated to better predict the gelling behavior of the tested plant proteins.

Overall, all the individual predictors showed a positive correlation (*p* < 0.05) with the predicted Gel (Figure S10). Solubility of the protein as a precondition for other functional properties including gelling was considered in modeling of Gel (Figure S10). Solubility of high molecular weight protein polymers, SP, was positively correlated with Gel. Such a relationship was inferred by previous researchers [12, 17, 19]. The WHC of proteins is another key indicator of Gel. WHC demonstrates the ability of the formed gel to entrap water and prevent syneresis. The extent of network formation in the gel governs its mechanical strength, as well as the ability to hold water [62]. Results confirmed a strong positive correlation between WHC and Gel (*p* < 0.05) (Figures 2e and S10), justifying the use of WHC a predictor for modeling Gel. Positive correlation between WHC and gel hardness for soy protein isolate has been demonstrated previously when using linear regression [62, 63]. Distinctively, in our study non‐linear interaction between WHC and Gel was also modeled using advanced non‐linear ML models.

Extent of denaturation as inferred by U, also positively affected Gel (Figures 2c and S10). Thermal denaturation of proteins leads to partial or complete unfolding of the protein molecules, exposing hydrophobic residues and sulfhydryl groups that promote intermolecular interactions leading to gel formation [59]. While Gel was positively correlated with U (Figure 2c), fully denatured and highly aggregated proteins lead to the formation of weak gels [9, 15]. For yellow peas, Lie‐Piang et al. [29] demonstrated that some ML models predicted higher gel strength for native proteins than fully denatured and aggregated proteins. Finally, protein secondary structure, namely *β*‐sheet content, βs, was also positively correlated with Gel. A relatively high abundance of βs allows proteins to form organized gel networks as demonstrated for soy protein [64], pennycress and pea proteins [17], owing to their available high surface area for crosslinking [65].

Overall, observations highlighted the intricate relation among the variables (Sol, WHC, U, SP, βs) governing Gel, which were collectively utilized for predicting the gelling behavior of the tested proteins. However, there were some discrepancies between the predicted results (Figure S9) and the actual dataset for Gel. These discrepancies could be improved in future studies by including more data points. Nevertheless, notable differences in Gel for individual proteins may have arisen due to their molecular characteristics. For example, soy proteins possess higher levels of cysteine residues in their 11S glycinin compared to pea 11S legumin [17], potentially yielding stronger gels due to intermolecular disulfide linkages. Similarly, due to higher abundance of 11S legumins in chickpea protein compared to pea protein [19], chickpea protein showed higher gel strength, comparable to that of soy protein. Meanwhile, the relatively low gel strength of pennycress protein could be attributed to its relatively low number of cysteine residues in its 11S cruciferin compared to soy 11S glycinin [57], and to its high napin (2S) to cruciferin (11S) ratio [9]. These findings highlighted the importance of composition and structure of proteins in determining gelation properties, underscoring the potential of using these predictors in ML models to predict complex gelling behavior of plant proteins and design protein‐based gels with optimal characteristics.

### 3.3 Implications of Machine Learning Models for Practical Applications

The ML model predictions were notably affected by the individual protein species that were investigated, potentially due to several tested and not‐tested unique compositions, molecular configurations, amino acid sequences, and other protein structural and functional properties, showing distinct patterns in the model predictions. Interestingly, regardless of model types, the predictions mostly compare well with the analytical data. Therefore, modeling of protein functionality by relying on a few, yet impactful, structural characteristics as independent variables can help with decision‐making in the food industry. Without performing all structure and functionality assays, individual protein functional properties can be predicted using computational models. Overall, determination of independent variables such as SH, ZP, U, WHC, SP, and βs can accurately predict protein solubility, emulsifying properties, and gelation. Therefore, accurate determination of these predictors could lead to accurate prediction of functionality. In terms of decision making related to the selection of ingredients with desired functionality for a targeted application, models predicting highest solubility, for instance, can be used in the design of high protein beverages, concentrates/shakes, milk/cream analogs. On the other hand, models predicting good emulsifying properties (EAI, EC) can be used for the formulations of food emulsions such as mayonnaise, salad dressing, ice cream alternatives, frozen desserts, coffee whiteners, and cake batters. Lastly, models predicting strong gelation properties can be used in designing plant‐based egg, meat, cheese, and yogurt analogues. Such a decision‐making and model‐based selection can be conducted for the best performing model, which was *Gaussian* based Support Vector Regression model in this study. The model features and codes can be implemented in the coding platform to predict the functionality of plant proteins from an unseen new dataset. The following code snippet can be used for predicting targeted functionality (Y), using the model predictors ($X_{i}$), for any unseen data.

gaussian_svr_model < − svm(Y~data = train_data, kernel = “radial”, cost = 10, gamma = 1).

saveRDS(gaussian_svr_model, file = “gaussian_svr_model.rds”).

predictions < − predict(gaussian_svr_model, newdata = new_data).

Trained ML models can aid in tailored applications and optimization of formulations using different plant protein ingredients. However, further research is warranted to explore additional predictors, use advanced ML algorithms, and a higher number of datasets, thus improving the power of prediction.

## 4 Conclusion

Plant proteins have gained significant attention as functional food ingredients, with a wide variety of applications such as beverages, meat and dairy analogues, and emulsified food systems. The species, cultivars, and processing methods of different plat proteins govern their structural characteristics, which in turn play a pivotal role in determining their functional properties and behavior in various food systems. In this study, ML algorithms have been trained and tested on a plant protein database to quantify the complex relationship between protein structure and functionality. The attempt was to identify the characteristics (surface hydrophobicity, zeta potential, undenatured protein content, soluble protein polymer content, and *β*‐sheet content) that best correlate with and predict the protein functionality (solubility, emulsifying activity index, emulsifying capacity, and gel strength). The *Gaussian* based Support Vector Regression model performed the best and most accurately predicted solubility ($R^{2}$ = 0.8906), emulsifying activity index ($R^{2}$ = 0.7383), emulsifying capacity ($R^{2}$ = 0.7978), and gel strength ($R^{2}$ = 0.8822), while ensuring that the predictions did not violate any physical constraints. This suggests that the model has a high probability of success for predicting functional properties using unseen datasets. These results demonstrated, for the first time, the effective prediction of plant protein functional properties using only a few macromolecular structural characteristics, without the need for time‐consuming experiments as well as measurement of the properties of individual proteins (or fractionated and purified proteins). Our approach is valuable for screening or quality control purposes. In practice, manufacturers might measure a subset of physical properties (e.g., surface hydrophobicity) as quick indicators of functionality, especially when high‐throughput functional testing isn't feasible. Also, we tested various models and found one of the models to be the best among them. While limiting the need of extensive analytical characterization, this approach can streamline the selection and optimization of plant proteins for various food formulations, leading to a more efficient and informed decision‐making in terms of food ingredient selection. Future research direction could include expanding the dataset to include a wider range of plant protein sources, cultivars, and their processing/extraction methods. Such modeling can thus be used to select cultivars and processing conditions for the production of the most functional protein ingredients. Additionally, the incorporation of other structural predictors and the exploration of more advanced ML models like convolutional neural networks (CNN) will further enhance the accuracy of the predictions. With the development in ML, the predictive models will serve as indispensable tools in the selection of plant‐based functional ingredients for product design and other sectors of the food industry.

## Author Contributions

**Ronit Mandal:** conceptualization, methodology, investigation, formal analysis, visualization, writing – original draft, review, and editing. **Sara Malvar:** writing – reviewing and editing. **Ranveer Chandra:** writing – reviewing and editing. **Baraem P. Ismail:** conceptualization, supervision, project administration, writing – review and editing, funding acquisition.

## Funding

The authors acknowledges the support of the Microsoft Research team. The work was funded by Microsoft Corporation.

## Conflicts of Interest

The authors declare no conflicts of interest.

## Supporting information


**Figure S1:** Box plot distributions showing variations in the studied plant protein solubility, emulsifying activity index, emulsifying capacity, and gel strength.

**Figure S2:** Box‐plot showing distribution of predicted protein solubility (pH 7.0) (Sol) based on different machine learning models (*n* = 41). Individual species of proteins are color coded as per legend.

**Figure S3:** Residual plot showing residuals for different predicted functional properties based on the best fit model. Solubility (Sol) (a), emulsifying activity (EAI) (b), emulsifying capacity (EC) (c), and gel strength (Gel) (d) fitted to *Gaussian* support vector regression model.

**Figure S4:** Predicted protein solubility (pH 7.0) (Sol), using selected feasible models, as a function of individual predictors fitted to different machine learning models. The predictors, in different colors as shown in the legend, have been normalized to make the scale similar for comparison.

**Figure S5:** Box‐plot showing distribution of predicted protein emulsifying activity index (EAI) based on different machine learning models (*n* = 41). Individual species of proteins are color coded as per legend.

**Figure S6:** Box‐plot showing distribution of predicted protein emulsifying capacity (EC) based on different machine learning models (*n* = 43). Individual species of proteins are color coded as per legend.

**Figure S7:** Predicted protein emulsifying activity index (EAI) as a function of individual predictors fitted to different machine learning models. The predictors have been normalized for make the scale same for their comparison.

**Figure S8:** Predicted protein emulsifying capacity (EC) as a function of individual predictors fitted to different machine learning models. The predictors have been normalized for make the scale same for their comparison.

**Figure S9:** Box‐plot showing distribution of predicted protein gel strength (Gel) based on different machine learning models (*n* = 24). Individual species of proteins are color coded as per legend.

**Figure S10:** Predicted protein gel strength (Gel) as a function of individual predictors fitted to different machine learning models. The predictors have been normalized for make the scale same for their comparison.

**Data S1:** prot70130‐sup‐0011‐Tables.docx.

## Data Availability

The data that support the findings of this study are openly available in Zenodo at https://doi.org/10.5281/zenodo.17637762.
