# Supplementary material for: A Machine Learning Approach to Predict Functional Performance From Measurable Protein Structural Characteristics: A Screening Tool for Protein Ingredient Quality
Source: Proteins. 2026 Mar 11;94(8):1458–84. doi: 10.1002/prot.70130 (PMC13327453; doi:10.1002/prot.70130)
Supplement: Supplementary file 1 — Figure S1: Box plot distributions showing variations in the studied plant protein solubility, emulsifying activity index, emulsifying capacity, and gel strength. [file PROT-94-1458-s004.pptx]

## Slide 1
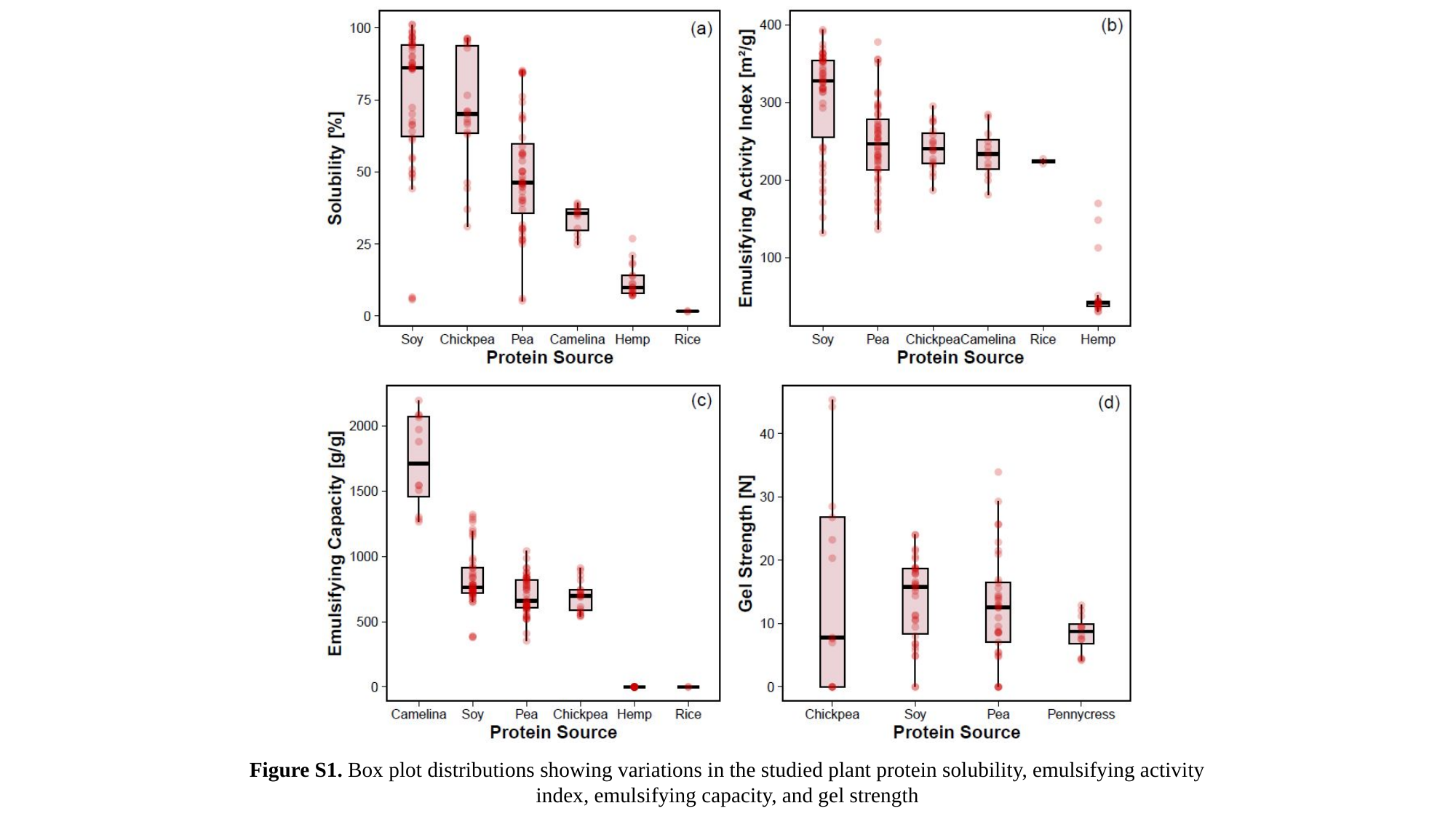

Figure S1. Box plot distributions showing variations in the studied plant protein solubility, emulsifying activity index, emulsifying capacity, and gel strength
