# Supplementary figures and images for: A Machine Learning Approach to Predict Functional Performance From Measurable Protein Structural Characteristics: A Screening Tool for Protein Ingredient Quality
Source: Proteins. 2026 Mar 11;94(8):1458–84. doi: 10.1002/prot.70130 (PMC13327453; doi:10.1002/prot.70130)

## Slide 1
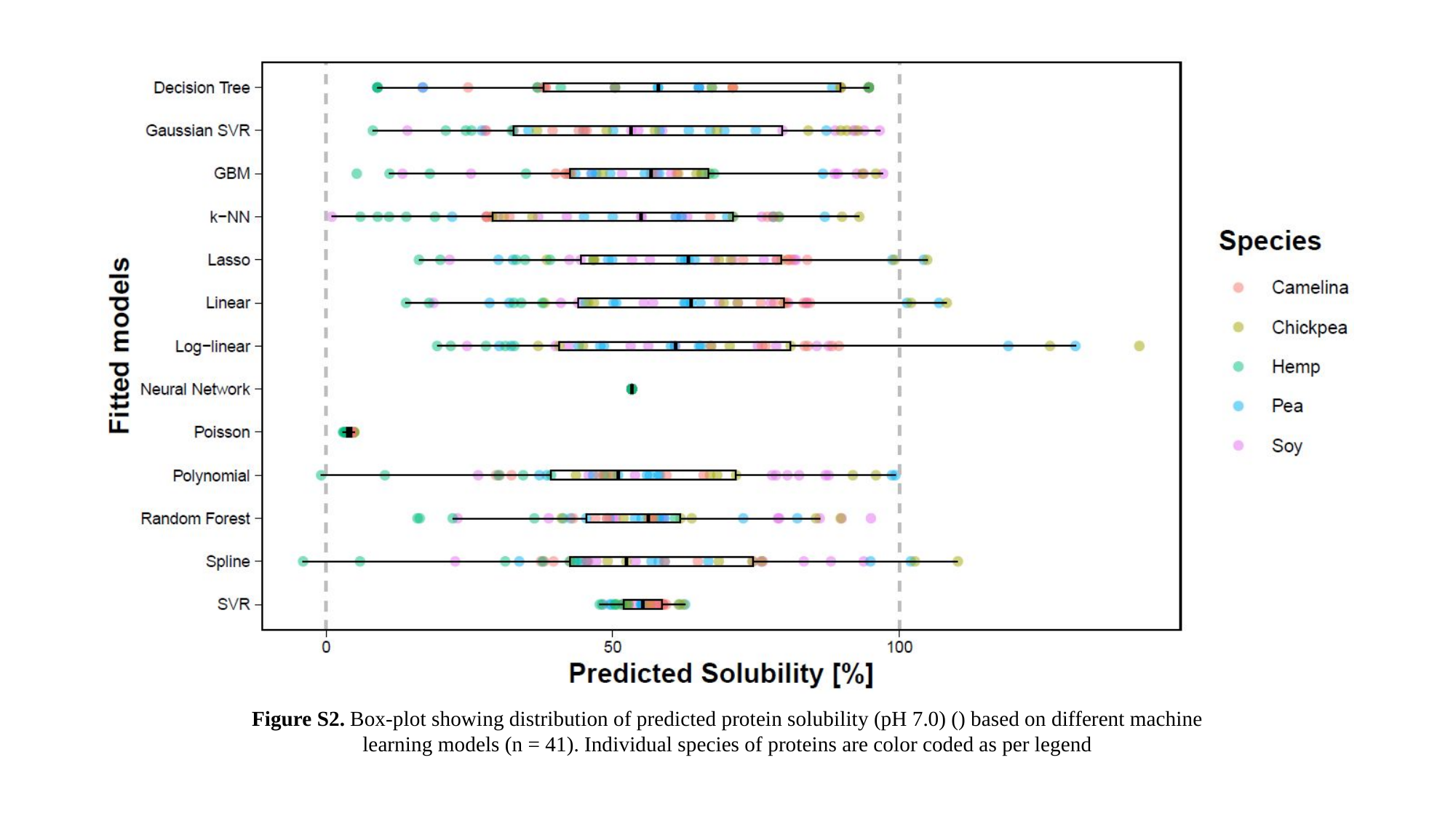

Supplement: Supplementary file 2 — Figure S2: Box‐plot showing distribution of predicted protein solubility (pH 7.0) (Sol) based on different machine learning models (n = 41). Individual species of proteins are color coded as per legend. [file PROT-94-1458-s010.pptx]

## Slide 1
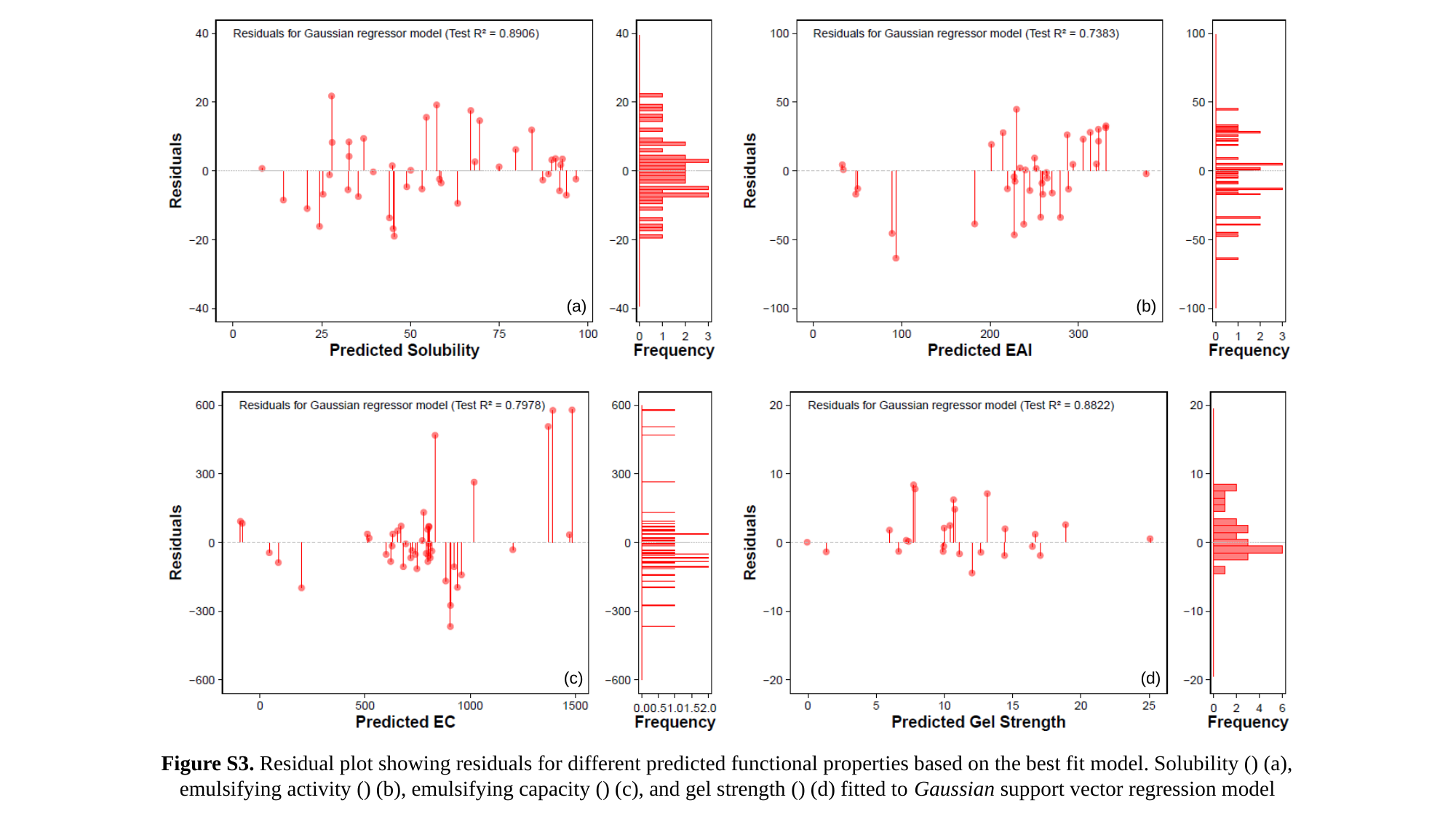

(a)
(b)
(c)
(d)

Supplement: Supplementary file 3 — Figure S3: Residual plot showing residuals for different predicted functional properties based on the best fit model. Solubility (Sol) (a), emulsifying activity (EAI) (b), emulsifying capacity (EC) (c), and gel strength (Gel) (d) fitted to Gaussian support vector regression model. [file PROT-94-1458-s006.pptx]

## Slide 1
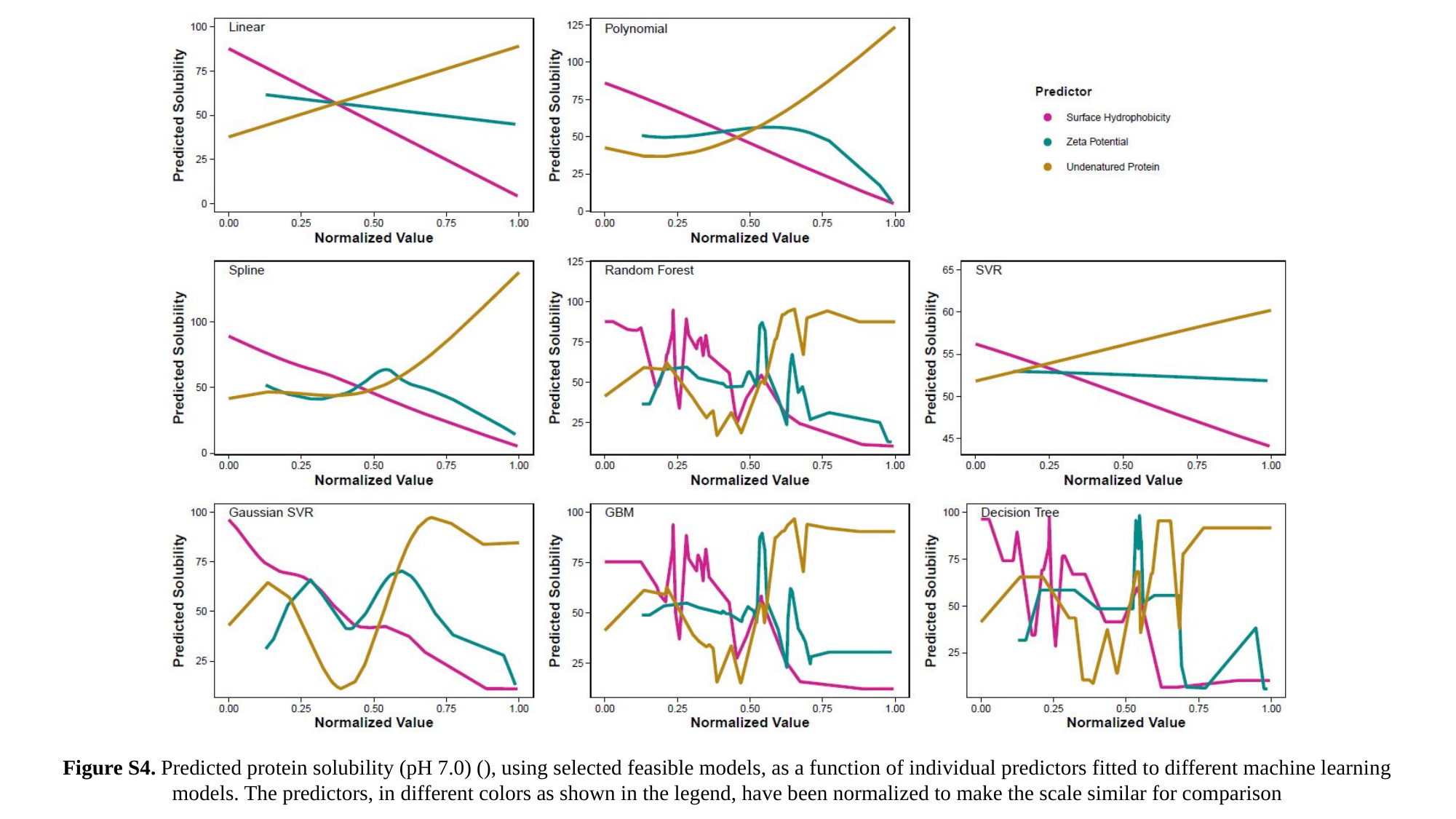

Supplement: Supplementary file 4 — Figure S4: Predicted protein solubility (pH 7.0) (Sol), using selected feasible models, as a function of individual predictors fitted to different machine learning models. The predictors, in different colors as shown in the legend, have been normalized to make the scale similar for comparison. [file PROT-94-1458-s011.pptx]

## Slide 1
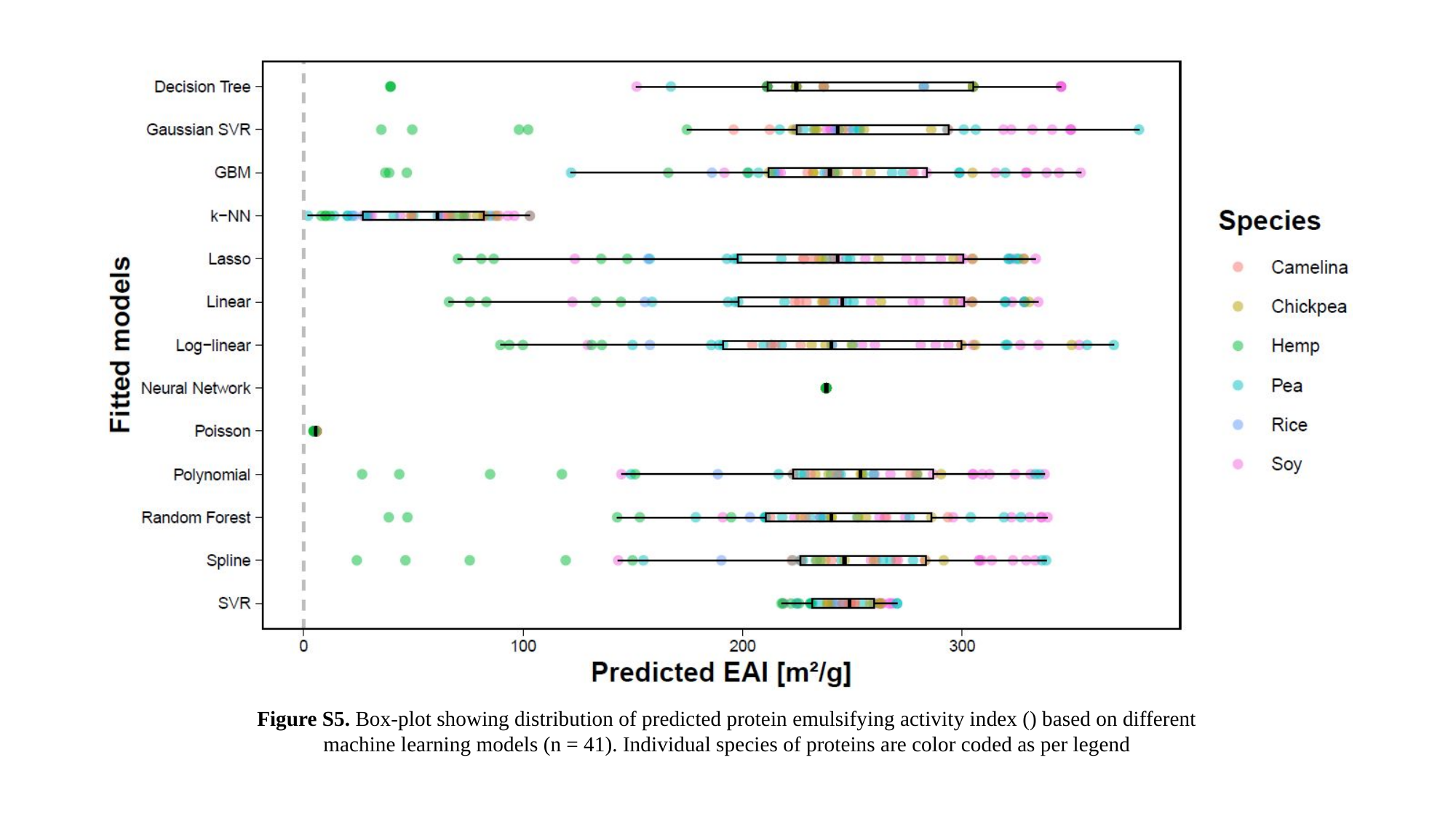

Supplement: Supplementary file 5 — Figure S5: Box‐plot showing distribution of predicted protein emulsifying activity index (EAI) based on different machine learning models (n = 41). Individual species of proteins are color coded as per legend. [file PROT-94-1458-s005.pptx]

## Slide 1
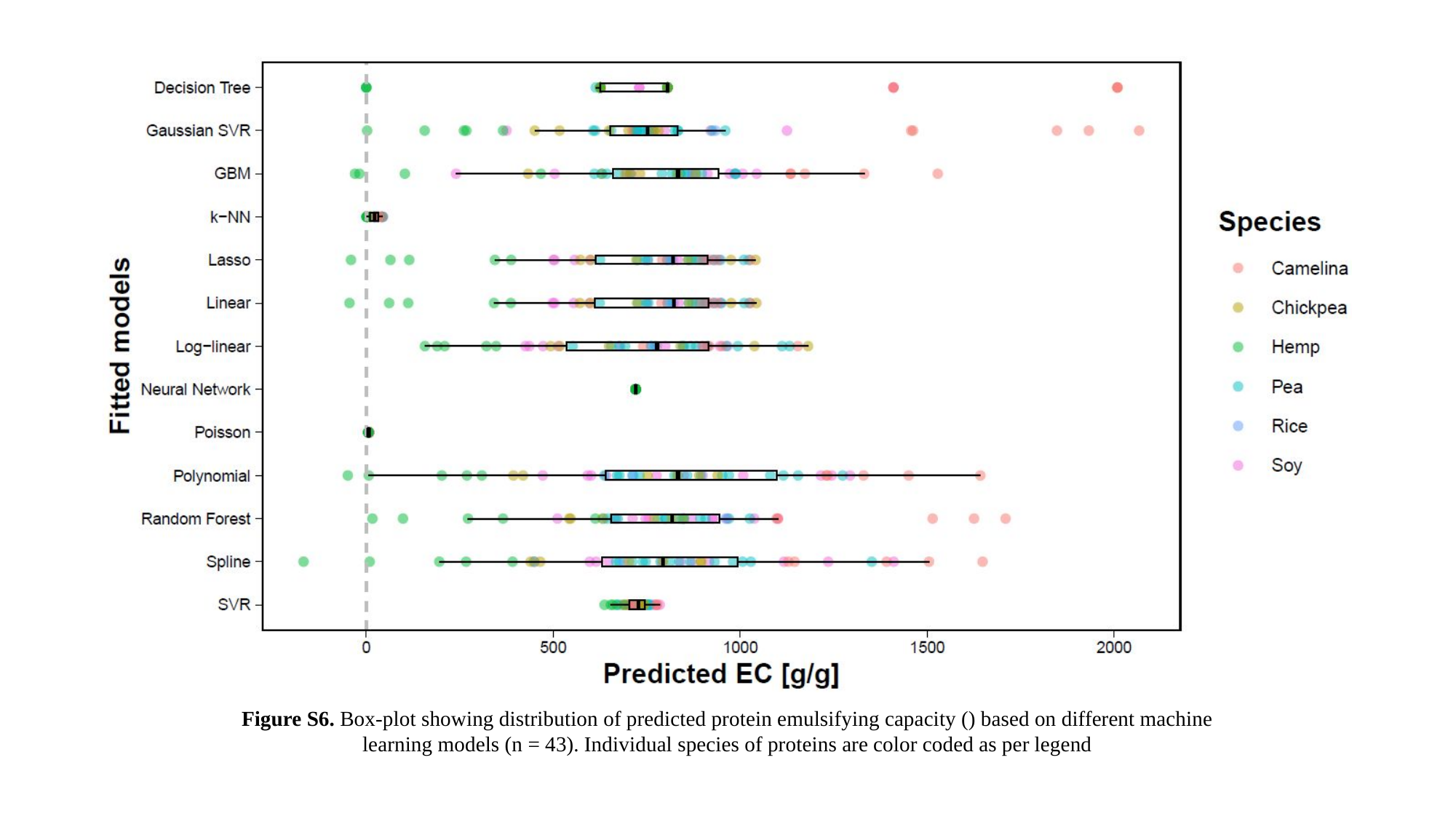

Supplement: Supplementary file 6 — Figure S6: Box‐plot showing distribution of predicted protein emulsifying capacity (EC) based on different machine learning models (n = 43). Individual species of proteins are color coded as per legend. [file PROT-94-1458-s009.pptx]

## Slide 1
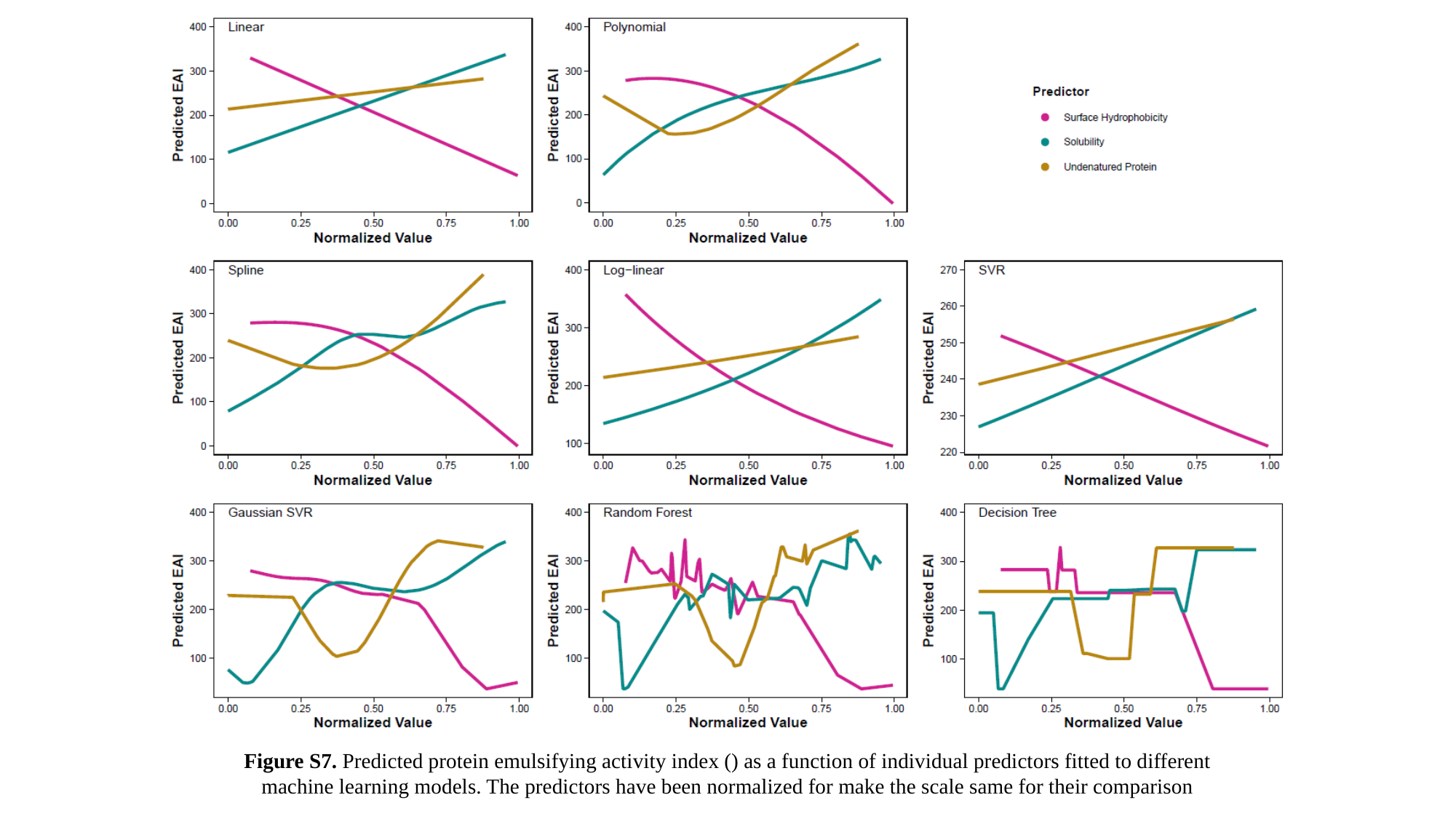

Supplement: Supplementary file 7 — Figure S7: Predicted protein emulsifying activity index (EAI) as a function of individual predictors fitted to different machine learning models. The predictors have been normalized for make the scale same for their comparison. [file PROT-94-1458-s008.pptx]

## Slide 1
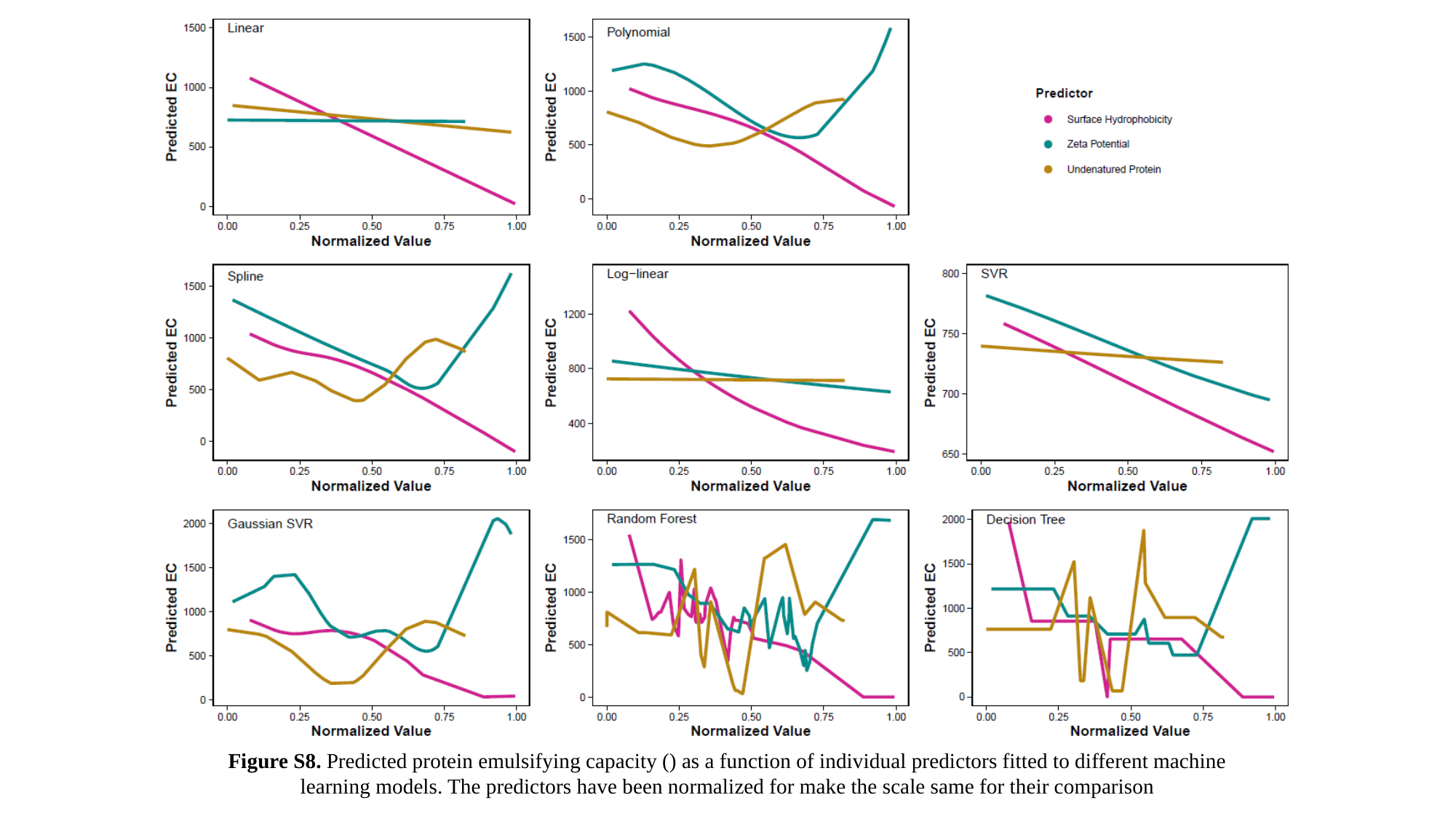

Supplement: Supplementary file 8 — Figure S8: Predicted protein emulsifying capacity (EC) as a function of individual predictors fitted to different machine learning models. The predictors have been normalized for make the scale same for their comparison. [file PROT-94-1458-s002.pptx]

## Slide 1
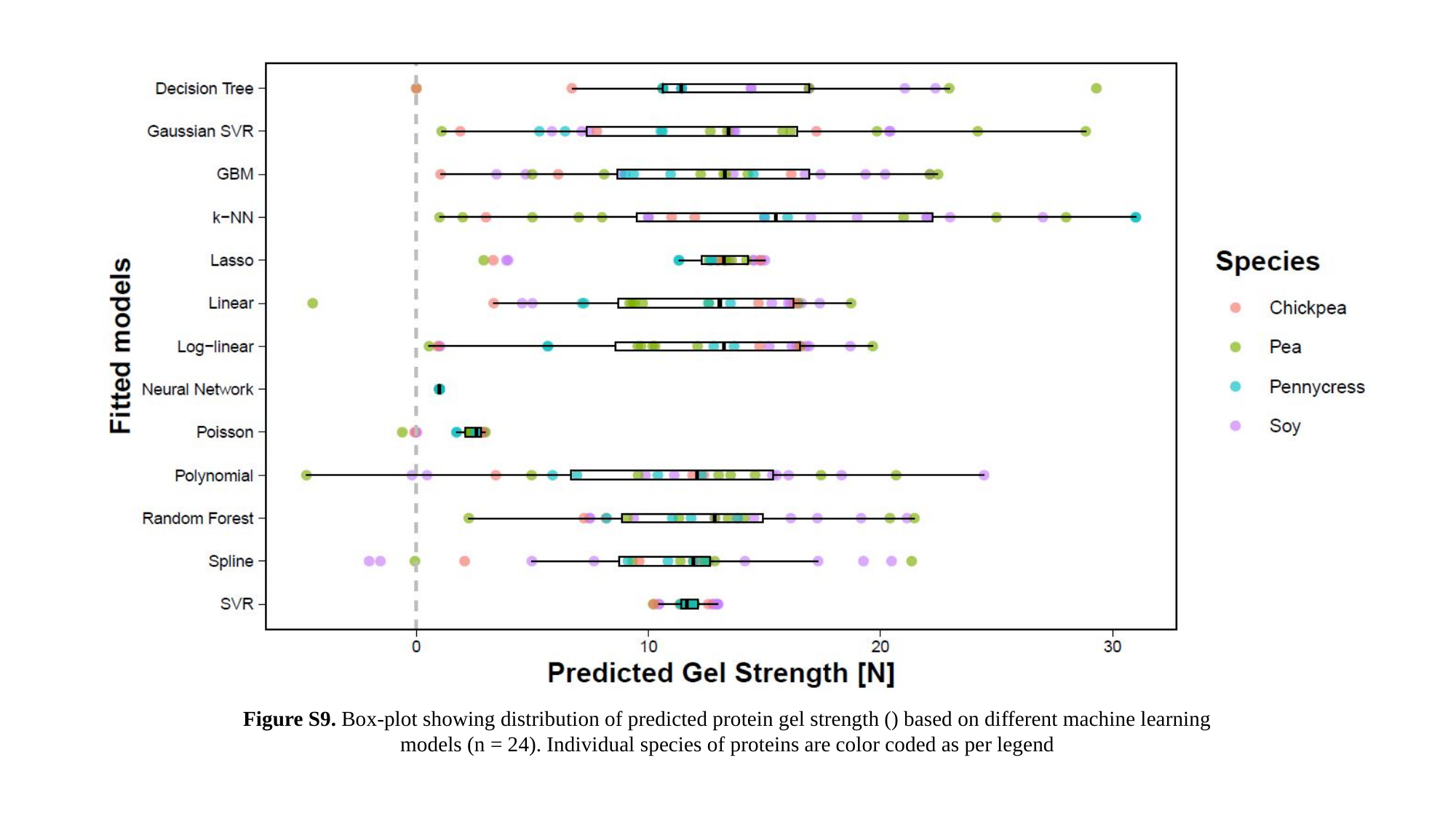

Supplement: Supplementary file 9 — Figure S9: Box‐plot showing distribution of predicted protein gel strength (Gel) based on different machine learning models (n = 24). Individual species of proteins are color coded as per legend. [file PROT-94-1458-s003.pptx]

## Slide 1
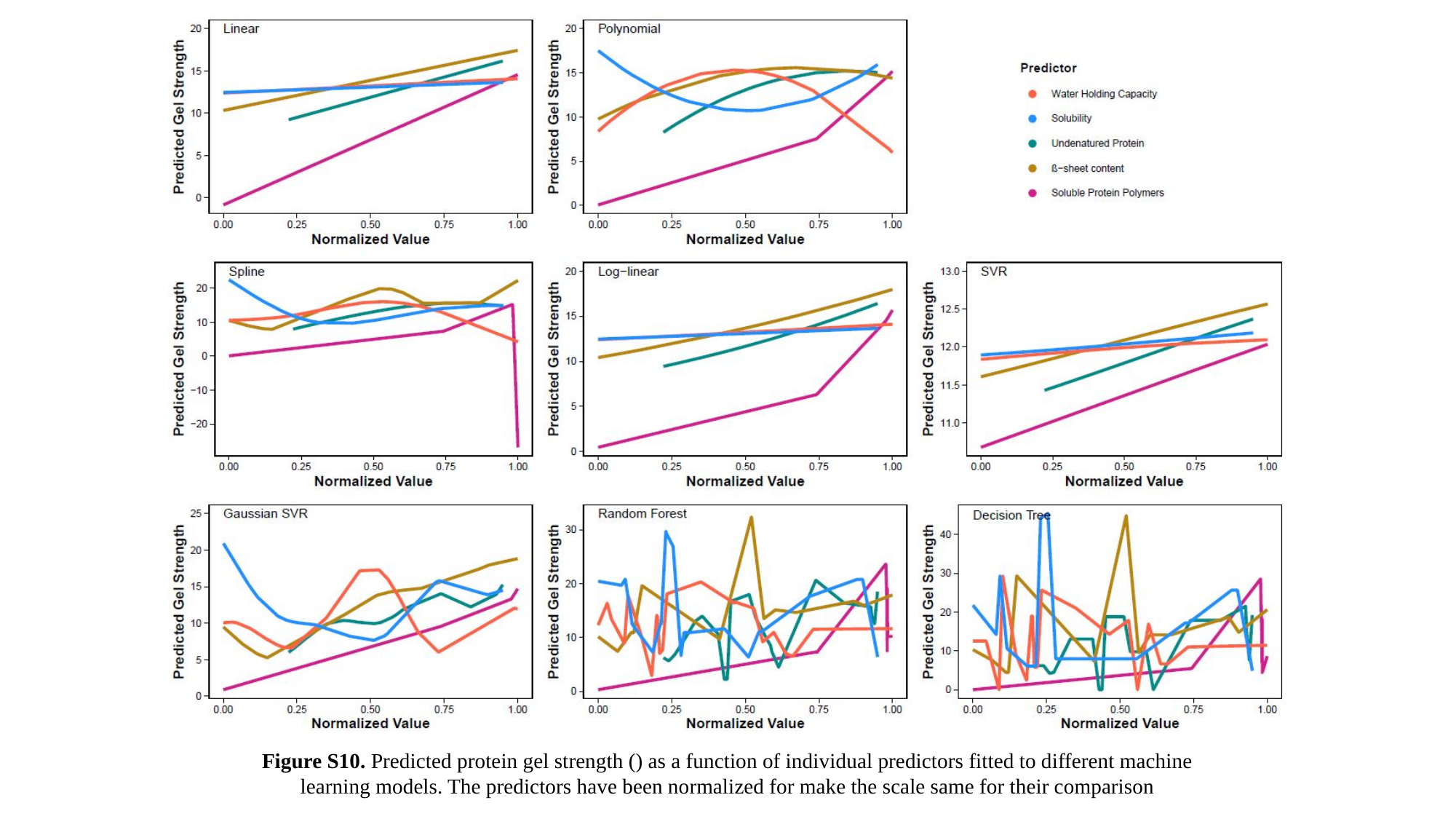

Supplement: Supplementary file 10 — Figure S10: Predicted protein gel strength (Gel) as a function of individual predictors fitted to different machine learning models. The predictors have been normalized for make the scale same for their comparison. [file PROT-94-1458-s007.pptx]
