# Supplementary material for: A Machine Learning Approach to Predict Functional Performance From Measurable Protein Structural Characteristics: A Screening Tool for Protein Ingredient Quality
Source: Proteins. 2026 Mar 11;94(8):1458–84. doi: 10.1002/prot.70130 (PMC13327453; doi:10.1002/prot.70130)
Supplement: Supplementary file 11 — Data S1: prot70130‐sup‐0011‐Tables.docx. [file PROT-94-1458-s001.docx]

**Supplemental Information**

**for**

**A machine learning approach to predict functional performance from measurable protein structural characteristics: a screening tool for protein ingredient quality**


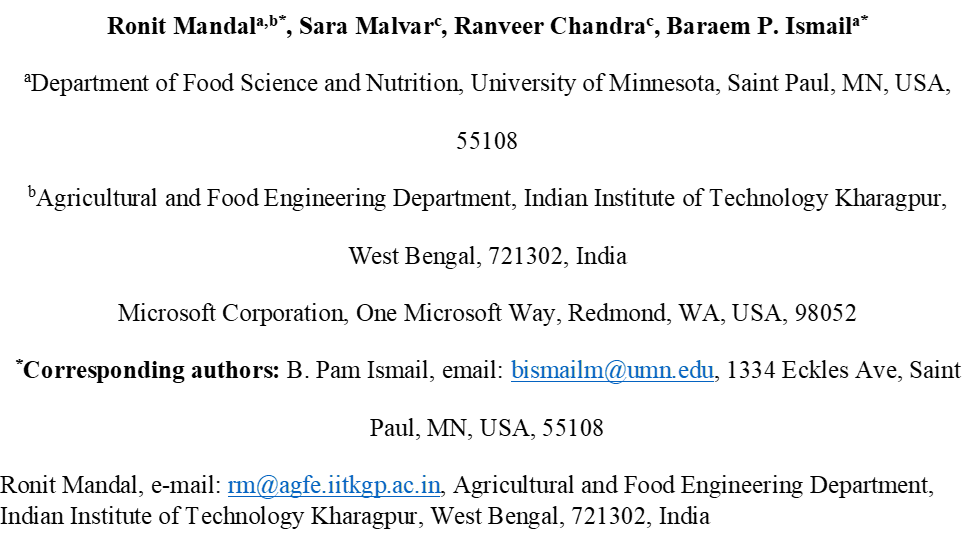


**Table S1: Hyperparameter tuning for the different machine learning models used in the study.**

| **Models** | **Solubility** | **Emulsifying Activity Index** | **Emulsification Capacity** | **Gel Strength** |
| --- | --- | --- | --- | --- |
| Polynomial | Degree (RMSE)  1(22.36)  2 (15.32)  **3 (14.25)**  4 (18.50) | Degree (RMSE)  1 (59.68)  2 (55.57)  **3 (54.33)**  4 (55.43) | Degree (RMSE)  1 (403.5)  2 (317.0)  3 (307.2)  **4 (306.6)** | Degree (RMSE)  1 (7.671)  **2 (6.857)**  3 (6.981)  4 (8.353) |
| Random Forest | mtry (OOBError)  **1 (226.4)**  2 (240.5)  3 (231.7) | mtry (OOBError)  **1 (2185)**  2 (2424)  3 (2627) | mtry (OOBError)  **1 (57445)**  2 (62833)  3 (65299) | mtry (OOBError)  1 (45.42)  2 (43.35)  **4 (42.82)** |
| Support Vector Regression (SVR) | Cost, Gamma (RMSE)  **0.1, 0.01 (23.35)**  1, 0.01 (24.82)  10, 0.01 (24.58) | Cost, Gamma (RMSE)  **0.1, 0.01 (61.60)**  1, 0.01 (62.27)  10, 0.01 (62.27) | Cost, Gamma (RMSE)  **0.1, 0.01 (427.4)**  1, 0.01 (428.7)  10, 0.01 (428.9) | Cost, Gamma (RMSE)  **0.1, 0.01 (8.567)**  1, 0.01 (8.610)  10, 0.01 (8.609) |
| Gaussian SVR | Cost, Gamma (RMSE)  0.1, 0.01 (26.11)  1, 0.01 (21.10)  0.1, 0.1 (19.03)  0.1, 1 (17.10)  1, 1 (10.31)  **10, 1 (9.584)** | Cost, Gamma (RMSE)  0.1, 0.01 (63.15)  1, 0.01 (57.43)  0.1, 0.1 (54.34)  0.1, 1 (65.24)  1, 1 (47.02)  **10, 1 (**47.02**)** | Cost, Gamma (RMSE)  0.1, 0.01 (250.4)  1, 0.01 (437.1)  0.1, 0.1 (433.3)  0.1, 1 (434.94 )  1, 1 (241.8)  **10, 1 (**229.5**)** | Cost, Gamma (RMSE)  0.1, 0.01 (9.161)  1, 0.01 (8.554)  0.1, 0.1 (8.868)  0.1, 1 (8.909)  1, 1 (6.025)  **10, 1 (**3.871**)** |
| Spline | DF1, DF2, DF3 (RMSE)  5, 4, 3 (15.66)  3, 5, 3 (14.76)  **4, 5, 3 (14.68)**  5, 5, 3 (14.83)  3, 3, 4 (15.86) | DF1, DF2, DF3 (RMSE)  4, 4, 3 (55.45)  5, 4, 3 (55.69)  **3, 5, 3 (53.50)**  4, 5, 3 (54.65)  5, 5, 3 (54.83) | DF1, DF2, DF3 (RMSE)  3, 4, 5 (289.7)  4, 4, 5 (288.8)  **5, 4, 5 (288.6)**  3, 5, 5 (289.7)  4, 5, 5 (288.9) | DF1, DF2, DF3, DF4, DF5 (RMSE)  5, 5, 4, 3, 3 (33.76)  3, 3, 5, 3, 3 (6.734)  **4, 3, 5, 3, 3 (6.703)**  5, 3, 5, 3, 3 (6.840)  3, 4, 5, 3, 3 (16.97) |
| Neural Network | Hidden unit, Decay (RMSE )  5, 0.01 (28.70) | Hidden unit, Decay (RMSE )  5, 0.01 (186.0) | Hidden unit, Decay (RMSE )  5, 0.01 (495.5) | Hidden unit, Decay (RMSE )  5, 0.01 (6.857) |
| k-Nearest Neighbors (k-NN) | k (RMSE)  1 (28.66)  2 (24.95)  3 (25.19)  4 (24.31)  5 (24.13)  6 (24.26)  7 (23.72)  **8 (23.44)**  9 (23.50)  10 (23.58) | k (RMSE)  1 (85.58)  2 (75.68)  3 (72.62)  4 (67.61)  5 (63.24)  6 (63.64)  7 (63.19)  8 (62.35)  9 (61.88)  **10 (61.36)** | k (RMSE)  1 (396.3)  2 (361.0)  3 (345.6)  4 (332.7)  5 (322.3)  6 (318.9)  7 (317.0)  8 (317.2)  **9 (313.4)**  10 (319.3) | k (RMSE)  **1 (5.594)**  2 (6.576)  3 (7.757)  4 (7.922)  5 (8.111)  6 (7.958)  7 (7.995)  8 (8.181)  9 (8.252)  10 (8.195) |
| Decision Tree | Maximum Depth, Minimum Samples for Split, Minimum Samples in Leaf, CP (RMSE)  2, 2, 1, 0.001 23.00)  4, 2, 1, 0.001 (20.74)  **6, 2, 1, 0.001 (19.84)**  2, 5, 1, 0.001 23.00)  4, 5, 1, 0.001 (21.94) | Maximum Depth, Minimum Samples for Split, Minimum Samples in Leaf, CP (RMSE)  6, 10, 4, 0.001 (64.66)  2, 2, 1, 0.01 (62.02)  4, 2, 1, 0.01 (58.08)  **6, 2, 1, 0.01 (54.66)**  2, 5, 1, 0.01 (62.02) | Maximum Depth, Minimum Samples for Split, Minimum Samples in Leaf, CP (RMSE)  6, 10, 4, 0.001 (315.6)  2, 2, 1, 0.01 (346.8)  **4, 2, 1, 0.01 (238.8)**  6, 2, 1, 0.01 (297.5)  2, 5, 1, 0.01 (346.8) | Maximum Depth, Minimum Samples for Split, Minimum Samples in Leaf, CP (RMSE)  4, 2, 1, 0.001 (6.355)  **6, 2, 1, 0.001 (5.350)**  2, 5, 1, 0.001 (7.012)  4, 5, 1, 0.001 (6.355)  6, 5, 1, 0.001 (5.839) |
| Gradient Boosting Machine (GBM) | Number of trees = 100,  Interaction depth = 7,  Shrinkage = 0.1,  Minimum number of observations (samples) required in a node to be further split = 5 | Number of trees = 50,  Interaction depth = 7,  Shrinkage = 0.1,  Minimum number of observations (samples) required in a node to be further split = 5 | Number of trees = 50,  Interaction depth = 7,  Shrinkage = 0.1,  Minimum number of observations (samples) required in a node to be further split = 5 | Number of trees = 100,  Interaction depth = 7,  Shrinkage = 0.1,  Minimum number of observations (samples) required in a node to be further split = 5 |

RMSE = Root mean squared error

Values in bold shows the best outcome for tuned hyperparameters

**Table S2: Pearson’s correlation coefficient values and significance of relation between predicted functional properties of plant proteins and individual predictors based on different machine learning models**

| **Model** | **Predicted functional properties** | **Predictors** |
| --- | --- | --- |
| Linear Regression | Solubility | Surface Hydrophobicity [*r* = 1.000; *P* = 0.0000*]  Zeta Potential [*r* = 1.000; *P* = 0.0000]  Undenatured protein [*r* = 1.000; *P* = 0.0000] |
|  | Emulsifying activity index | Surface Hydrophobicity [*r* = 1.000; *P* = 0.0000]  Solubility [*r* = 1.000; *P* = 0.0000]  Undenatured protein [*r* = 1.000; *P* = 0.0000] |
|  | Emulsifying capacity | Surface Hydrophobicity [*r* = 1.000; *P* = 0.0000]  Zeta Potential [*r* = 1.000; *P* = 0.0000]  Undenatured protein [*r* = 1.000; *P* = 0.0000] |
|  | Gel strength | Water Holding Capacity [*r* = 1.000; *P* = 0.0000]  Solubility [*r* = 1.000; *P* = 0.0000]  Undenatured protein [*r* = 1.000; *P* = 0.0000]  β-sheet content [*r* = 1.000; *P* = 0.0000]  Soluble protein polymers [*r* = 1.000; *P* = 0.0000] |
| Polynomial Regression | Solubility | Surface Hydrophobicity [*r* = 0.9998; *P* = 0.0000]  Zeta Potential [*r* = 0.6232; *P* = 1.352×10^-5^]  Undenatured protein [*r* = 0.8532; *P* = 0.0000] |
|  | Emulsifying activity index | Surface Hydrophobicity [*r* = 0.9424; *P* = 0.0000]  Solubility [*r* = 0.9672; *P* = 0.0000]  Undenatured protein [*r* = 0.3676; *P* = 0.01806] |
|  | Emulsifying capacity | Surface Hydrophobicity [*r* = 0.9773; *P* = 0.0000]  Zeta Potential [*r* = 0.2021; *P* = 0.2050]  Undenatured protein [*r* = 0.1460; *P* = 0.3623] |
|  | Gel strength | Water Holding Capacity [*r* = 0.9845; *P* = 0.0000]  Solubility [ *r* = 0.9232; *P* = 0.0000]  Undenatured protein [ *r* = 0.9194; *P* = 0.0000]  β-sheet content [*r* = 0.0204; *P* = 0.9248]  Soluble protein polymers [*r* = 0.2152; *P* = 0.3125] |
| Random Forest | Solubility | Surface Hydrophobicity [*r* = 0.0805; *P* = 0.0000]  Zeta Potential [*r* = 0.4318; *P* = 0.005]  Undenatured protein [*r* = 0.6599; *P* = 2.688×10^-6^] |
|  | Emulsifying activity index | Surface Hydrophobicity [*r* = 0.8711; *P* = 0.0000]  Solubility [*r* = 0.8050; *P* = 0.0000]  Undenatured protein [*r* = 0.3054; *P* = 0.0522] |
|  | Emulsifying capacity | Surface Hydrophobicity [*r* = 0.6972; *P* = 4.101×10^-7^]  Zeta Potential [*r* = 0.03835; *P* = 0.8118]  Undenatured protein [*r* = 0.1056; *P* = 0.5111] |
|  | Gel strength | Water Holding Capacity [*r* = 0.8139; *P* = 1.316×10^-6^]  Solubility [*r* = 0.5202; *P* = 0.0092]  Undenatured protein [*r* = 0.5787; *P* = 0.0031]  β-sheet content [*r* = 0.1342; *P* = 0.5320]  Soluble protein polymers [*r* = 0.1041; *P* = 0.6284] |
| Support Vector Machine Regression (SVR) | Solubility | Surface Hydrophobicity [*r* = 0.9998; *P* = 0.0000]  Zeta Potential [*r* = 0.9951; *P* = 0.0000]  Undenatured protein [*r* = 0.9999; *P* = 0.0000] |
|  | Emulsifying activity index | Surface Hydrophobicity [*r* = 0.99993; *P* 0.0000]  Solubility [*r* = 0.99996; *P* = 0.0000]  Undenatured protein [*r* = 0.99997; *P* = 0.0000] |
|  | Emulsifying capacity | Surface Hydrophobicity [*r* = 0.99994; *P* = 0.0000]  Zeta Potential [*r* = 0.9986; *P* = 0.0000]  Undenatured protein [*r* = 0.9996; *P* = 0.0000] |
|  | Gel strength | Water Holding Capacity [*r* = 0.9999; *P* = 0.0000]  Solubility [*r* = 0.9999; *P* = 0.0000]  Undenatured protein [*r* = 0.9999; *P* = 0.0000]  β-sheet content [*r* = 0.9938; *P* = 0.0000]  Soluble protein polymers [*r* = 0.9994; *P* = 0.0000] |
| Gaussian SVR | Solubility | Surface Hydrophobicity [*r* = 0.9885; *P* = 0.0000]  Zeta Potential [*r* = 0.2750; *P* = 0.0819]  Undenatured protein [*r* = 0.5925; *P* = 4.476×10^-5^] |
|  | Emulsifying activity index | Surface Hydrophobicity [*r* = 0.9086; *P* = 0.0000]  Solubility [*r* = 0.8646; *P* = 0.0000]  Undenatured protein [*r* = 0.3631; *P* = 0.0189] |
|  | Emulsifying capacity | Surface Hydrophobicity [*r* = 0.8367; *P* = 0.0000]  Zeta Potential [*r* = 0.1265; *P* = 0.4306]  Undenatured protein [*r* = 0.3095; *P* = 0.4897] |
|  | Gel strength | Water Holding Capacity [*r* = 0.9978; *P* = 0.0000]  Solubility [*r* = 0.9432; *P* = 0.0000]  Undenatured protein [*r* = 0.8861; *P* = 8.477×10^-9^]  β-sheet content [*r* = 0.3778; *P* = 0.0687]  Soluble protein polymers [*r* = 0.0493; *P* = 0.8191] |
| Spline Regression | Solubility | Surface Hydrophobicity [*r* = 0.9993; *P* = 0.0000]  Zeta Potential [*r* = 0.5248; *P* = 4.280×10^-4^]  Undenatured protein [*r* = 0.8060; *P* = 0.0000] |
|  | Emulsifying activity index | Surface Hydrophobicity [*r* = 0.9361; *P* = 0.0000]  Solubility [*r* = 0.9568; *P* = 0.0000]  Undenatured protein [*r* = 0.3279; *P* = 0.0364] |
|  | Emulsifying capacity | Surface Hydrophobicity [*r* = 0.9723; *P* = 0.0000]  Zeta Potential [*r* = 0.1445; *P* = 0.3674]  Undenatured protein [*r* = 0.1067; *P* = 0.5069] |
|  | Gel strength | Water Holding Capacity [*r* = 0.1842; *P* = 0.3890]  Solubility [*r* = 0.0725; *P* = 0.7363]  Undenatured protein [*r* = 0.8661; *P* = 4.527×10^-8^]  β-sheet content [*r* = 0.8999; *P* =2.177×10^-9^  Soluble protein polymers [*r* = 0.4597; *P* = 0.0238] |
| Decision Tree | Solubility | Surface Hydrophobicity [*r* = 0.7699; *P* = 4.009×10^-9^]  Zeta Potential [*r* = 0.4064; *P* = 0.0084]  Undenatured protein [*r* = 0.5437; *P* = 2.388×10^-4^] |
|  | Emulsifying activity index | Surface Hydrophobicity [*r* = 0.8582; *P* = 0.0000]  Solubility [*r* = 0.8352; *P* = 0.0000]  Undenatured protein [*r* = 0.2652; *P* = 0.0938] |
|  | Emulsifying capacity | Surface Hydrophobicity [*r* = 0.6726; *P* = 1.456×10^-6^]  Zeta Potential [*r* = 0.1443; *P* = 0.3679]  Undenatured protein [*r* = 0.0094; *P* = 0.9534] |
|  | Gel strength | Water Holding Capacity [*r* = 0.7096; *P* = 1.301×10^-4^]  Solubility [*r* = 0.4891; *P* = 0.0153]  Undenatured protein [*r* = 0.4090; *P* = 0.0472]  β-sheet content [*r* = 0.1159; *P* = 0.5898]  Soluble protein polymers [*r* = 0.0849; *P* = 0.6933] |
| Poisson | Solubility | Surface Hydrophobicity [*r* = 1.000; *P* = 0.0000]  Zeta Potential [*r* = 1.000; *P* = 0.0000]  Undenatured protein [*r* = 1.000; *P* = 0.0000] |
|  | Emulsifying activity index | Surface Hydrophobicity [*r* = 1.000; *P* = 0.0000]  Solubility [*r* = 1.000; *P* = 0.0000]  Undenatured protein [*r* = 1.000; *P* = 0.0000] |
|  | Emulsifying capacity | Surface Hydrophobicity [*r* = 1.000; *P* = 0.0000]  Zeta Potential [*r* = 1.000; *P* = 0.0000]  Undenatured protein [*r* = 1.000; *P* = 0.0000] |
|  | Gel strength | Water Holding Capacity [*r* = 1.000; *P* = 0.0000]  Solubility [*r* = 1.000; *P* = 0.0000]  Undenatured protein [*r* = 1.000; *P* = 0.0000]  β-sheet content [*r* = 1.000; *P* = 0.0000]  Soluble protein polymers [*r* = 1.000; *P* = 0.0000] |
| Gradient Boosting Machine (GBM) | Solubility | Surface Hydrophobicity [*r* = 0.8041; *P* = 0.0000]  Zeta Potential [*r* = 0.3543; *P* = 0.0230]  Undenatured protein [*r* = 6765; *P* = 1.201×10^-4^] |
|  | Emulsifying activity index | Surface Hydrophobicity [*r* = 0.8567; *P* = 0.0000]  Solubility [*r* = 0.8804; *P* = 0.0000]  Undenatured protein [*r* = 0.2819; *P* = 0.0741] |
|  | Emulsifying capacity | Surface Hydrophobicity [*r* = 0.8128; *P* = 0.0000]  Zeta Potential [*r* = 0.2246; *P* = 0.1581]  Undenatured protein [*r* = 0.1187; *P* = 0.4595] |
|  | Gel strength | Water Holding Capacity [*r* = 0.7471; *P* = 2.740×10^-5^]  Solubility [*r* = 0.6106; *P* = 0.0015]  Undenatured protein [*r* = 0.5592; *P* = 0.0045]  β-sheet content [*r* = 0.1349; *P* = 0.5296]  Soluble protein polymers [*r* = 0.0221; *P* = 0.9182] |
| Log-Linear Regression | Solubility | Surface Hydrophobicity [*r* = 0.9624; *P* = 0.0000]  Zeta Potential [*r* = 0.9986; *P* = 0.0000]  Undenatured protein [*r* = 0.9914; *P* = 0.0000] |
|  | Emulsifying activity index | Surface Hydrophobicity [*r* = 0.9840; *P* = 0.0000]  Solubility [*r* = 0.9930; *P* = 0.0000]  Undenatured protein [*r* = 0.9934; *P* = 0.0000] |
|  | Emulsifying capacity | Surface Hydrophobicity [*r* = 0.9735; *P* = 0.0000]  Zeta Potential [*r* = 0.9989; *P* = 0.0000]  Undenatured protein [*r* = 0.99999; *P* = 0.0000] |
|  | Gel strength | Water Holding Capacity [*r* = 0.9687; *P* = 0.0000]  Solubility [*r* = 0.9971; *P* =0.0000]  Undenatured protein [*r* = 0.9979; *P* = 0.0000]  β-sheet content [*r* = 0.9998; *P* = 0.0000]  Soluble protein polymers [*r* = 0.9999; *P* = 0.0000] |

*r* = Correlation coefficient; *P* = probability value

*0.0000 denotes *P* values < 1.000×10^-9^

**Figures S1-S10: Submitted as individual files.**

**Figure S1.** Box plot distributions showing variations in the studied plant protein solubility (a), emulsifying activity index (b), emulsifying capacity (c), and gel strength (d)

**Figure S2.** Box-plot showing distribution of predicted protein solubility (pH 7.0) ($Sol$) based on different machine learning models (n = 41). Individual species of proteins are color coded as per legend

**Figure S3.** Residual plot showing residuals for different predicted functional properties based on the best fit model. Solubility ($Sol$) (a), emulsifying activity index ($EAI$) (b), emulsifying capacity ($EC$) (c), and gel strength ($Gel$) (d) fitted to *Gaussian* based Support Vector Regression model

**Figure S4.** Predicted protein solubility (pH 7.0) ($Sol$), using selected models, as a function of individual predictors fitted to different machine learning models. The predictors, in different colors as shown in the legend, have been normalized to make the scale similar for comparison

**Figure S5.** Box-plot showing distribution of predicted protein emulsifying activity index ($EAI$) based on different machine learning models (n = 41). Individual species of proteins are color coded as per legend

**Figure S6.** Box-plot showing distribution of predicted protein emulsifying capacity ($EC$) based on different machine learning models (n = 41). Individual species of proteins are color coded as per legend

**Figure S7.** Predicted protein emulsifying activity index ($EAI$), using selected models, as a function of individual predictors fitted to different machine learning models. The predictors, in different colors as shown in the legend, have been normalized to make the scale similar for comparison

**Figure S8.** Predicted protein emulsifying capacity ($EC$), using selected models, as a function of individual predictors fitted to different machine learning models. The predictors, in different colors as shown in the legend, have been normalized to make the scale similar for comparison

**Figure S9.** Box-plot showing distribution of predicted protein gel strength ($Gel$) based on different machine learning models (n = 24). Individual species of proteins are color coded as per legend

**Figure S10.** Predicted protein gel strength ($Gel$), using selected models, as a function of individual predictors fitted to different machine learning models. The predictors, in different colors as shown in the legend, have been normalized to make the scale similar for comparison
